# Monooxygenase- and Dioxygenase-Catalyzed Oxidative Dearomatization of Thiophenes by Sulfoxidation, *cis*-Dihydroxylation and Epoxidation

**DOI:** 10.3390/ijms23020909

**Published:** 2022-01-14

**Authors:** Derek R. Boyd, Narain D. Sharma, Paul J. Stevenson, Patrick Hoering, Christopher C. R. Allen, Patrick M. Dansette

**Affiliations:** 1 School of Chemistry and Chemical Engineering, Queen’s University of Belfast, Belfast BT9 5AG, UK; n.sharma@qub.ac.uk (N.D.S.); p.stevenson@qub.ac.uk (P.J.S.); 2 School of Biological Sciences, Queen’s University of Belfast, Belfast BT9 5AJ, UK; patrick@hoering.com (P.H.); c.allen@qub.ac.uk (C.C.R.A.); 3 Laboratoire de Chimie et Biochimie Pharmacologiques et Toxicologiques, UMR CNRS 8601, Université de Paris, 45 rue des Saints-Pères, 75006 Paris, France; patrick.dansette@u-paris.fr

**Keywords:** thiophene, thiophene epoxide, thiophene *S*-oxide, S-oxide dimer, thiophene *cis*- and *trans*-dihydrodiols, thiophene hydrate, dioxygenase, monooxygenase, cytochrome P450, drug metabolism

## Abstract

Enzymatic oxidations of thiophenes, including thiophene-containing drugs, are important for biodesulfurization of crude oil and drug metabolism of mono- and poly-cyclic thiophenes. Thiophene oxidative dearomatization pathways involve reactive metabolites, whose detection is important in the pharmaceutical industry, and are catalyzed by monooxygenase (sulfoxidation, epoxidation) and dioxygenase (sulfoxidation, dihydroxylation) enzymes. Sulfoxide and epoxide metabolites of thiophene substrates are often unstable, and, while *cis*-dihydrodiol metabolites are more stable, significant challenges are presented by both types of metabolite. Prediction of the structure, relative and absolute configuration, and enantiopurity of chiral metabolites obtained from thiophene enzymatic oxidation depends on the substrate, type of oxygenase selected, and molecular docking results. The racemization and dimerization of sulfoxides, *cis*/*trans* epimerization of dihydrodiol metabolites, and aromatization of epoxides are all factors associated with the mono- and di-oxygenase-catalyzed metabolism of thiophenes and thiophene-containing drugs and their applications in chemoenzymatic synthesis and medicine.

## 1. Introduction

**Scheme 1:**
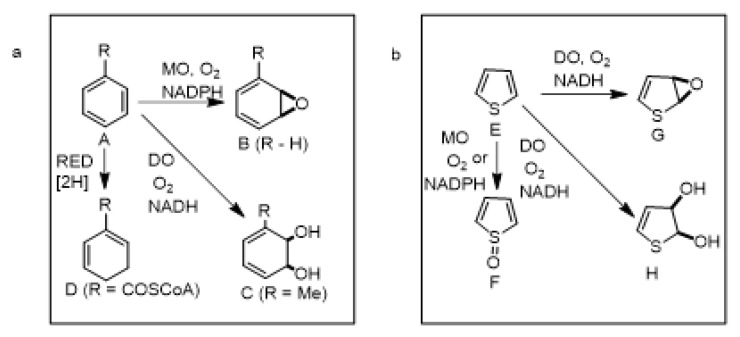
(**a**) Dearomatization of arenes (**A**). (**b**) Dearomatization of thiophene (**E**).

Although the link between aromaticity and resulting molecular stability is well established, enzymes have a remarkable capacity for dearomatization of many stable and recalcitrant arene and heteroarene substrates. Enzyme-catalyzed dearomatization reactions have been reported under both oxidative and reductive conditions. Examples of redox enzymatic dearomatization reactions of benzene rings A (R = H, Me, COSCoA) that give conjugated cyclohexadiene metabolites include: (i) monooxygenase (MO)-catalyzed epoxidation to yield an arene oxide B (R = H) [1], (ii) ring-hydroxylating dioxygenase (DO)-catalyzed *cis* dihydroxylation to yield a *cis*-dihydrodiol C (R = Me) [2], (iii) reductase (CoARed)-catalyzed reduction to give a dihydroarene D (R = COSCoA) (Scheme 1a) [3]. 

Attempted chemical approaches to the oxidative dearomatization reactions of carbocyclic arenes, shown in Scheme 1a, often result in further oxidation, or rearomatization of the initial products. Due to their instability, relatively few arene oxide metabolites **B** have been isolated from monooxygenase (MO)-catalyzed epoxidations of substituted benzene substrates **A**. Ring-hydroxylating dioxygenase (DO)-catalyzed *cis*-dihydroxylation of similar substrates **A** can, however, produce more stable *cis*-dihydrodiols **C**. Despite their limited stability, *cis*-dihydrodiol metabolites have been widely used in the chemoenzymatic synthesis of natural products and other metabolites [4,5,6,7,8,9]. 

The ability of similar oxidative dearomatization reactions of a thiophene substrate **E** to replicate MO-catalyzed epoxidation to yield epoxide **G**, DO-catalyzed *cis*-dihydroxylation to give *cis*-dihydrodiol **H**, and heteroatom oxidation to give sulfoxide **F** (Scheme 1b) using either type of oxygenase enzyme is examined in this review. 

Aromaticity of the monocyclic six-membered arenes, e.g., benzene, and five-membered heteroarenes, e.g., pyrrole, thiophene, and furan, confers extra stability and depends on the descriptor used [10]. Resonance energies have been widely used as an indicator of decreasing aromaticity in a sequence, from the most stable benzene > thiophene > pyrrole > furan. Most other qualitative and quantitative descriptors of aromaticity also suggest that the sequence should be: benzene > thiophene > pyrrole > furan **[11]**.

As acceptable thiophene substrates for ring-hydroxylating dioxygenases are generally smaller than for monooxygenase substrates, the topic of dioxygenase-catalyzed *cis*-dihydroxylation and sulfoxidation of thiophene substrates is initially considered. The only *cis*-dihydrodiol metabolites from the monocyclic five-membered heteroarenes, thiophene, pyrrole, and furan, of sufficient stability to be isolated and characterized are derived from thiophene substrates. Thus, the relatively stable *cis*-dihydrodiol metabolite **H** was obtained from toluene dioxygenase (TDO)-catalyzed oxidation of thiophene **E** along with the unstable thiophene-*S*-oxide **F** (Scheme 1b) [12]. 

Thiophenes and sulfur-containing polycyclic aromatic hydrocarbons (thiaarenes), present in the aromatic fraction of crude oil, include monocyclic thiophenes (e.g., dialkyl thiophenes), bicyclic thiaarenes (e.g., benzothiophenes), and tricyclic thiaarenes (e.g., dibenzothiophenes, naphthothiophenes) [13,14]. The requirement to remove these organosulfur constituents of crude oil stimulated interest in their oxidative biodegradation by *Pseudomonas* bacteria (biodesulfurization), as shown by the extensive studies of the Fedorak group [15,16,17,18,19,20,21,22,23,24,25]. Evidence of enzymatic dearomatization of thiophenes, to yield 2,3-dione, sulfoxide, sulfoxide dimer, and sulfone metabolites, was supplied by GC-MS and GC-FTIR analyses.

Early studies of the bacterial metabolism of thiaarenes also showed that dioxygenase-catalyzed *cis*-dihydroxylation of carbocyclic and heterocyclic rings could yield chiral *cis*-dihydrodiols as transient metabolites. Furthermore, isolation of these intermediates was possible when using *P. putida* mutant strains (or *E. coli* recombinant strains), expressing dioxygenases where an enzyme that is usually present in the metabolic pathway, a *cis*-dihydrodiol dehydrogenase was blocked (or absent) [26,27,28]. 

Biotransformations of monocyclic thiophene and polycyclic thiophene (thiaarene) substrates were conducted using wild-type, mutant, and recombinant bacterial strains expressing ring-hydroxylating dioxygenases with different active site capacities. The most widely used enzymes were toluene dioxygenase (TDO), naphthalene dioxygenase (NDO), and biphenyl dioxygenase (BPDO). The exact nature of the catalytic cycle involved in ring-hydroxylating dioxygenase-catalyzed *cis*-dihydroxylations remains unresolved, but possible mechanisms for producing arene metabolites are available [29,30]. 

This review deals with the stability, structure, stereochemistry, and mechanism involved during dioxygenase- and monooxygenase-catalyzed oxidative metabolism and dearomatization of mono- and poly-cyclic thiophenes via sulfoxidation*, cis*-dihydroxylation, and epoxidation. Ring-hydroxylating dioxygenase enzymes generally oxidize only relatively small (mono- to penta-cyclic) arene substrates compared with monooxygenase enzymes that can catalyze oxidation of much larger and more highly substituted substrates. Where common dioxygenase- and monooxygenase-catalyzed sulfoxidations occur, the results are linked by cross-references. The possibility of lessons learned from dioxygenase metabolism of thiophenes, being of value in monooxygenase metabolism, is explored. 

## 2. Dioxygenase-Catalyzed Dearomatization of Thiophenes 1a–g 

### 2.1. Enzymatic Oxidation of Thiophenes ***1a***–***g*** to Yield cis-Dihydrodiols

The thienyl ring system is ubiquitous in the environment, with many monocyclic thiophenes being found as plant natural products and constituents of fossil fuels [13,31]. The bacterial biodegradation of thiophene **1a** [12] and alkyl-substituted thiophenes, [22,25,32] present in bitumen and crude oils, was examined as a route to biodesulfurization. 

Relatively few reports of dioxygenase-catalyzed *cis*-dihydroxylation of monocyclic thiophenes have been reported in the literature. BPDO-catalyzed dihydroxylation of 3-hexylthiophene yielded a dihydrodiol metabolite with an unknown stereoconfiguration and showed no evidence of sulfoxide bioproducts [32]. A study of the metabolites isolated from TDO- and NDO-catalyzed oxidations over a wider range of monocyclic thiophene substrates revealed *cis*-dihydrodiols, *cis*/*trans*-dihydrodiols, and sulfoxides as common chiral bioproducts (Section 2.1, Section 2.2, Section 2.3, Section 3.1 and Section 3.2) [12]. 

Enantiomeric excess (*ee*) values of thiophene *cis*-dihydrodiol metabolites were determined by chiral stationary phase HPLC and GC analyses [33,34]. An alternative method for enantiopure *cis*-dihydrodiol metabolites required NMR analysis of diMTPA esters formed by reactions of (+) and (−)-2-methoxy-2-(trifluoromethyl)phenylacetyl chloride (MTPA-Cl) [35] with 4-phenyl-1,2,4-triazoline-3,5-dione cycloadducts [36] or with hydrogenated *cis*-dihydrodiols [37]. 

NMR analyses of boronate diastereoisomers, formed by the reaction of thiophene *cis*-dihydrodiols with (+)- and (−)-[2-(-methoxyethyl)phenyl]boronic acid (MEPBA), provided both *ee* values and absolute configuration assignments [38,39,40]. Furthermore, X-ray crystallography of *cis*-dihydrodiols or their diMTPA derivatives [33,37,41], and comparison of electronic circular dichroism (ECD) spectra with density functional theory ECD spectra, gave unambiguous absolute configurations for thiophene *cis*-dihydrodiol metabolites [41,42,43].

**Scheme 2:**
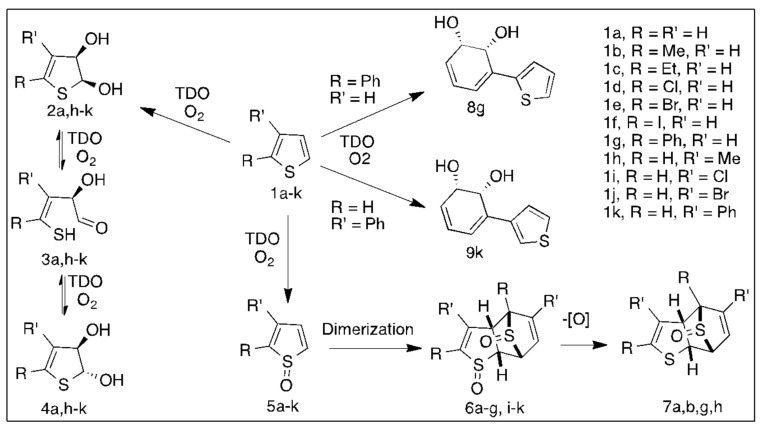
TDO-catalyzed metabolism of thiophenes **1a–k**.

Many enantiopure *cis*-dihydrodiol metabolites from arene and heteroarene substrates are used in the synthesis of natural products [4,5,6,7,8,9], including arene oxides [44]. The potential use of thiophene *cis*-dihydrodiols in the future chemoenzymatic synthesis of thiophene epoxide metabolites produced during monooxygenase-catalyzed oxidations is explored herein. The *cis*-dihydroxylation dearomatization of monocyclic thiophenes **1a,h–k**, using TDO, (*P.putida* UV4) yielded the corresponding *cis*-diol metabolites **2a**, **2h–k**, along with the *trans* isomers **4a**, **h–k** (Scheme 2, Table 1) [12]. The formation of *trans*-dihydrodiol **4a** was unusual, since only *cis*-dihydrodiol metabolites were formed from monocyclic arenes with TDO [4,5,6,7,8,9]. *trans*-Dihydrodiol metabolites of carbocyclic arenes generally result from monooxygenase-catalyzed epoxidation followed by epoxide hydrolase catalyzed hydrolysis. 

The hemithioacetal *cis*-isomer **2a** was the initially formed metabolite from thiophene **1a**; it spontaneously epimerized with the *trans* isomer **4a** via the undetected aldehyde intermediate **3a** (Scheme 2) [12]. The equilibrium ratio was found to be solvent-dependent, favoring *cis* isomer **2a** in CDCl_3_ (*ca*. 60%) and *trans*-isomer **4a** in more polar solvents CD_3_OD and D_2_O (>90%). A similar type of epimerization process involving aldehyde intermediates occurs during the mutarotation of *α*- and *β*-glucose. 

The *cis*-dihydrodiol metabolites, formed by TDO-catalyzed dihydroxylation, exclusively at the 2,3-bond of monosubstituted benzene substrates except for fluorobenzene, were enantiopure (>98% *ee*) [4,5,6,7,8,9]. Conversely, the isolated thiophene dihydrodiol metabolites were found to be mixtures of *cis*: *trans* isomers (**2a**:**4a**) and enantiomers (**2a**:**4a**, 44% *ee*) [12]. The lower *ee* values for dihydrodiols **2a** and **4a** could result from the reduced stereoselectivity due to weaker binding and more flexibility of the smaller heterocyclic ring **1a** within the TDO active site. Alternatively, the low *ee* value of *cis*-dihydrodiol **2a** could result from partial racemization of the aldehyde intermediate **3a**. Phenyl ring *cis*-hydroxylation of the larger 2-phenyl thiophene **1g** was also observed to yield 1*R*,2*S*-dihydrodiol **8g**, (>98% *ee*) (Table 1).

Biotransformations of mono- or poly-cyclic arenes and azaarenes, using *P. putida* mutant and *E. coli* recombinant strains, yielded only carbocyclic ring *cis*-dihydrodiol metabolites [36,45,46,47]. However, dioxygenase-catalyzed oxidation of monocyclic thiophenes **1b–1g** involved competing carbocyclic and heterocyclic ring *cis*-dihydroxylation and sulfoxidation pathways (Table 1). Replacement of phenylalanine 352 (Phe 352) by valine, within the NDO active site of *E. coli*-pDTG141, yielded *E. coli*-pF352V; this results in significant regio- and enantio-selectivity changes occurring during *cis*-dihydroxylation of polycyclic arenes [45,46]. A further marked change in regioselectivity is observed during NDO-catalyzed *cis*-dihydroxylation of 2-phenyl thiophene **1g**. One strain (*E. coli*-pFDTG141) yielded only a phenyl ring *cis*-dihydrodiol **8g**, while an alternative strain (*E. coli*-pF352V) gave thienyl ring *cis*:*trans* dihydrodiols (**2g**:**4g**) exclusively (Table 1). This shows the value of having different bacterial strains and dioxygenase enzymes available to produce preferred metabolites. 

### 2.2. Oxidations of Thiophenes ***1a***–***g*** to Yield Sulfoxide Metabolites

TDO- and NDO-catalyzed stereoselective sulfoxidations of alkylaryl sulfides using wild-type, mutant, recombinant strains and purified dioxygenase enzyme often yielded sulfoxide metabolites with high *ee* values [48,49,50]. Competition between *cis*-dihydroxylation and sulfoxidation of alkyl aryl sulfides and formation of *cis*-dihydrodiol sulfoxides was observed [51]. Results collected using recombinant strains expressing TDO and NDO provided supporting evidence that dioxygenases were also responsible for thiophene sulfoxidations, with both wild-type and mutant bacterial strains. A contest between sulfoxidation and epoxidation of monocyclic thiophenes and thiophene-containing drugs was also found using cytochrome P450 monooxygenases as biocatalysts (Section 6.2 and Section 6.3) [52,53,54,55,56,57,58].

Sulfoxidation of thiophenes **1a–g** was the major metabolic route with the *P. putida* UV4 strain (Scheme 2, Table 1). The main metabolite isolated from thiophene **1a** was sulfoxide dimer **6a** (Table 1) [12]. In addition, traces of the unstable sulfoxide **5a** were identified (GC-MS analysis) among other products. CYP-450 monooxygenase-catalyzed metabolism of thiophene **1a** and peroxyacid oxidation of thiophene **1a** yielded sulfoxide **5a**, which rapidly dimerized to form compound **6a [12]**

The monosulfoxide structure **5a** was both that of a cyclic diene and a dienophile, resulting in a spontaneous dimerization by Diels Alder cycloaddition to give the dimer **6a** (R = R′ = H). Further biotransformation via stereospecific deoxygenation yielded metabolite **7a** (Table 1, Scheme 2) [12]. Some reductases were found to catalyze preferential deoxygenation of alkylaryl sulfoxides and also the sterically more accessible sulfoxide group of dimer **6a** [59,60,61,62,63]. An unidentified sulfoxide reductase enzyme, expressed in *P. putida* UV4 [62] was particularly efficient. Methionine sulfoxide reductase-catalyzed deoxygenations of sulfoxides were also observed using *Pseudomonas* strains [59,60].

Sulfoxides formed by dioxygenase-catalyzed oxidation of alkylaryl sulfides [28,48,49,50] are configurationally stable, i.e., with inversion barriers (ΔG^‡^) > 23.0 kcal mol^−1^. Conversely, monocyclic thiophene sulfoxides (e.g., **1a** and **1g**) were predicted to have much lower barriers (ΔG^≠^ = 11–14 kcal mol^−1^), due to aromatic stabilization of their planar transition states [64,65,66]. Monocyclic thiophene sulfoxides, with bulky substituents, are more thermally stable. Thus 2,5-diphenylthiophene-1-oxide is sufficiently stable for X-ray crystallography studies [67]. Several racemic monocyclic thiophene sulfoxides, with bulky groups, were sufficiently stable to provide experimental confirmation of these relatively low barriers by NMR spectroscopic methods (ΔG^≠^ = 14.8; 15.9 kcal mol^−1^) [68,69]. Thiophene sulfoxide metabolites **5b–g** are susceptible to rapid racemization and cycloaddition, thus yielding racemic sulfoxide dimers **6b–g** (Scheme 2). Individual sulfoxide enantiomers can have different medical efficacy values, e.g., omeprazole, esomeprazole, modafinil, and armodafinil [70], but, with low sulfoxide inversion barriers for drugs containing monocyclic thiophenes, spontaneous racemization will occur. 

The results in Table 1 show that TDO-catalyzed sulfoxidation of thiophenes **1a–g** is the major dearomatization pathway, with *cis*-dihydroxylation of thienyl and phenyl rings, respectively, being minor pathways. CYP450-catalyzed oxidation of thiophenes **1a**, **1b**, and **1g** yielded the corresponding sulfoxides **6a**, **6b**, and **6g**, and their dimers **7a**, **7b**, and **7g**, as major products, but deoxygenation of dimers was not reported **(**Scheme 2). 

### 2.3. Molecular Docking of Thiophenes ***1a*** and ***1g*** at the TDO Active Site 

**Figure 1:**
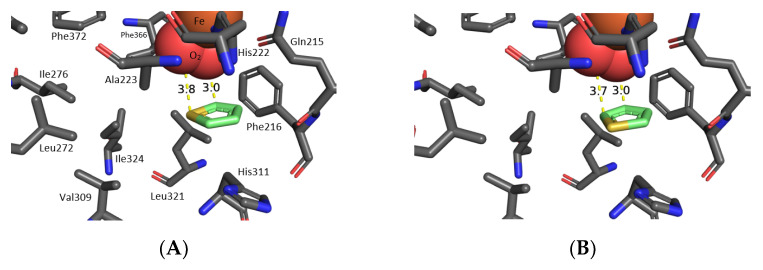
Preferred orientation of thiophene **1a** for TDO-catalyzed: sulfoxidation (**A**) and *cis*-dihydroxylation (**B**).

The Autodock Vina program was used for docking toluene **A** (R = Me, Scheme 1a) and other arene and heteroarene substrates within the active site of toluene dioxygenase [71,72]. An X-ray crystal structure of the TDO showed the toluene substrate bound at the active site by proximate amino acids but without dioxygen complexed to Fe(III) [73]. An X-ray crystal structure of NDO displayed dioxygen coordinated with Fe(III) and indole substrate bonded at the active site [74]. The O_2_-Fe(III) complex of NDO was then inserted into the TDO active site employing the reported procedure [72]. The interaction between dioxygen and Fe(III) during the catalysis of the *cis*-dihydroxylation is considered to involve a hydroperoxide (Fe-OOH) [29]. Attractive interactions between substrates and proximate amino acids (Phe-216, His-222, Ile-276, Leu-272, Ile-324, Val-309, Leu-272, Figure 1A) allow the preferred binding orientation to be predicted; it matched both with the regio-and stereo-selectivity observed during TDO-catalyzed *cis*-dihydroxylation of toluene and other substituted benzene substrates to form *cis*-dihydrodiols (Scheme 1a) [72,74,75,76,77,78,79]. 

**Figure 2:**
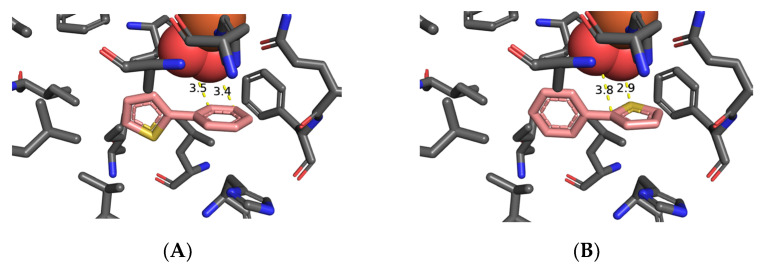
Preferred orientation of 2-phenylthiophene **1g** for TDO-catalyzed: *cis*-dihydroxylation (**A**) and sulfoxidation (**B**).

A similar in situ docking approach, applied earlier to other arene and heteroarene substrates [71,72], was used to determine the preferred binding orientations of thiophene **1a** (Figure 1A,B) and 2-phenylthiophene **1g** (Figure 2A,B). As expected for a smaller five-membered heteroarene substrate, binding energies (± 0.5 kcal mol^−1^) for thiophene **1a**, Figure 1A (−3.63 kcal mol^−1^) and B (−3.3 kcal mol^−1^), were lower compared to a six-membered arene substrate, e.g., toluene **A** (R = Me, −5.0 kcal mol^−1^). The minimum distances between the nearest oxygen and sulfur atoms (proximity value, 3.8 Å, Figure 1A), or between proximate oxygen atoms and the 2,3-bond (3.0–3.7 Å, Figure 1B), are similar to those found between TDO and toluene **A** (R = Me). The favored orientation of thiophene **1a** in Figure 1A is consistent with TDO-catalyzed sulfoxidation to yield sulfoxide **5a** and derived bioproducts **6a** and **7a [12]**. The reduced substrate size, lower binding energy, but favorable proximity factor for compound **1a** in Figure 1B could be factors in the reduced *ee* value (44%) and lower yield of the isolated heterocyclic *cis*/*trans* diols **2a**/**4a** (Table 1).

The preferred orientation of 2-phenylthiophene **1g** at the TDO active site (Figure 2A) has a relatively high binding energy (−5.5 kcal mol^−1^) and proximity of the phenyl group to dioxygen (3.4–3.5 Å). The predicted regioselectivity and absolute configuration of *cis*-dihydrodiol **8g** was found, experimentally, to be (1*S*,2*R*) (Table 1) [12]. 

The preferred orientation of substrate **1g**, at the TDO active site, predicted from the binding energy (−5.3 kcal mol^−1^) and the distance of the sulfur to dioxygen (2.9 Å), should yield (1S)-sulfoxide **5g**. However, the low inversion barrier, predicted for sulfoxide **5g** ΔG = 11.2 kcal mol^−1^) [64], indicated that it would rapidly racemize, and this was confirmed by the isolation of racemic dimer **6g** (Table 1). The docking of substrate **1g** did not present an orientation that would lead to *cis*-dihydrodiol metabolite **2g**, and none of the possible *cis*/*trans*-dihydrodiols **2b–g**/**4b–g** were isolated (Table 1) [12].

## 3. Dioxygenase-Catalyzed Dearomatization of 3-Substituted Thiophenes 1h–k

### 3.1. Oxidations of Thiophenes ***1h***–***k*** to Yield cis-Dihydrodiol and Sulfoxide Metabolites

Biotransformations (*P. putida* UV4) involving *cis*-dihydroxylations of 3-substituted thiophenes **1h–j** yielded, mainly, *cis*-dihydrodiols **2h–j** (Table 2) [12]. Spontaneous epimerization of metabolites **2h–j** gave mixtures of *cis*/*trans* dihydrodiols and enantiomers **2h**/**4h** (40–48% *ee*), **2i**/**4i** (49% *ee*), and **2j**/**4j** (44% *ee*). This very unusual observation merits rationalization since most *cis*-dihydrodiol metabolites from monosubstituted benzene substrates had very high *ee* values (>98%) [4,5,6,7,8,9]. It is proposed that the lower *ee* values found during TDO-catalyzed *cis*-dihydroxylation of thiophene **1a** and 3-substituted thiophenes **1h–j** could result from the reduced size of the substrates and increased flexibility within the TDO active site. TDO-catalyzed *cis*-dihydroxylation of the larger substrate **1k** yielded dihydrodiols **2k**/**4k** (>98% *ee*) and **9k** (>98% *ee*, Table 2).

Dioxygenase-catalyzed sulfoxidation was also an important dearomatization route for 3-substituted thiophenes **1h–k**, yielding racemic sulfoxide dimers **6i–k** from sulfoxide intermediates **5i–k** (Table 2). Dimer **6h** was undetected, as it was further metabolized via a stereoselective reductase-catalyzed partial deoxygenation to form monosulfoxide **7h** (51% *ee*) [12].

**Scheme 3:**
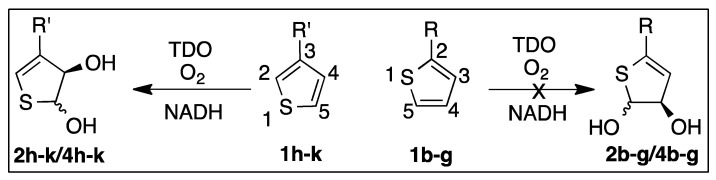
Contrasting TDO-catalyzed dihydroxylations for thiophene**s 1h–k** and **1b–g**.

The regioselectivity observed, during TDO-catalyzed *cis*-dihydroxylation of 3-substituted thiophenes **1h–k**, involved exclusive attack at the unsubstituted 4,5-bond of thiophenes **1h–k** to yield *cis*/*trans*-dihydrodiols **2h–k**/**4h–k** (Scheme 3) [12]. 

**Figure 3:**
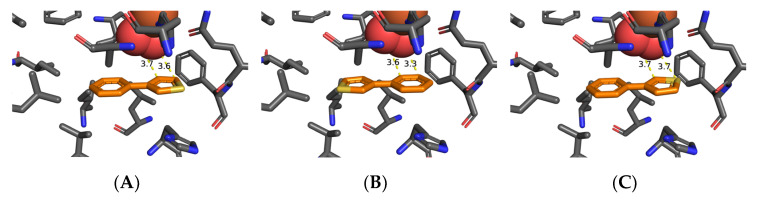
Preferred orientation of 3-phenylthiophene **1k** for TDO-catalyzed: thienyl *cis*-dihydroxylation (**A**), phenyl *cis*-dihydroxylation (**B**), and sulfoxidation (**C**).

Molecular docking studies (Figure 2A,B and Figure 3A–C) provided supporting evidence for the preferred regioselectivity found in Scheme 3. TDO-catalyzed dihydroxylation, at the 4,5-bond of thiophenes **1h–k**, is equivalent to *cis*-dihydroxylation exclusively at the 2,3-bond of monosubstituted benzene substrates (Scheme 1a). 

A remarkable change in chemoselectivity was observed during NDO-catalyzed oxidation of 3-phenyl thiophene **1k**; *cis*-hydroxylation of the phenyl ring gave diol **9k** (98**%** *E. coli* DTG 141) while oxidation of the thiophene ring produced sufoxide dimer **6k** (80% *E. coli* pF 353V, Table 2).

### 3.2. Molecular Docking of 3-Phenylthiophene ***1k*** at the TDO Active Site

The preferred orientation of 3-phenyl thiophene **1k** had a binding energy (−4.7 kcal mol^−1^) and proximity of dioxygen to the 4,5-bond of the thiophene ring (3.6–3.7 Å, Figure 3A). This would lead to *cis*-dihydroxylation and formation of *cis*-dihydrodiol **2k**, prior to equilibration with *trans*-dihydrodiol **4k**; it matched the experimental result (>98% *ee*, Table 2, Scheme 3) [12].

The orientation of substrate **1k** (Figure 3B), with a binding energy (−4.5 kcal mol^−1^), and proximity of the 2,3-bond of the phenyl ring to dioxygen (3.3–3.6 Å), would also lead to *cis*-dihydroxylation and the (1*S*,2*R*) absolute configuration of the isolated dihydrodiol **9k** (>98% *ee*, Table 2) [12]. The favored orientation of substrate **1k** (Figure 3C), with binding energy (−4.7 kcal mol^−1^), and proximity of the *S* atom to the nearest dioxygen atom (3.7 Å), would result in sulfoxidation of substrate **1k** to yield an excess of one enantiomer of 3-phenylthiophene-1-oxide **5k**, prior to its rapid racemization and dimerization to give disulfoxide **6k** (Table 2). Earlier GOLD and Autodock Vina molecular docking studies of mono- and poly-cyclic arene and heteroarene substrates within the TDO active site concluded that their preferred orientations were controlled by: (i) attractive edge-to-face (T-bond) interactions between orthogonal phenyl (Phe-216) groups of TDO and phenyl or pyridyl groups of substrates, (ii) van der Waals interactions with proximate hydrophobic amino acids (Ile-276, Leu-272, Ile-324, Val-309, Leu-272, and Phe-352, Figure 1A) [72,74,75,76,77,78,79]. Attractive edge-to-face interactions are also found between phenyl rings of TDO (Phe-216) and of substrates **1g** (Figure 2A) and **1k** (Figure 3B). Edge-to-face interactions, between phenyl and thienyl groups, were earlier identified by NMR and X-ray crystallographic studies of imines [80]. Similar novel edge-to-face interactions are observed between phenyl (Phe-216) and thienyl rings in substrates **1a** (Figure 1A,B), **1g** (Figure 2A,B), **1k** (Figure 3A–C).

## 4. Dioxygenase-Catalyzed Dearomatization of Benzo[*b*]thiophenes 10a–d

**Scheme 4:**
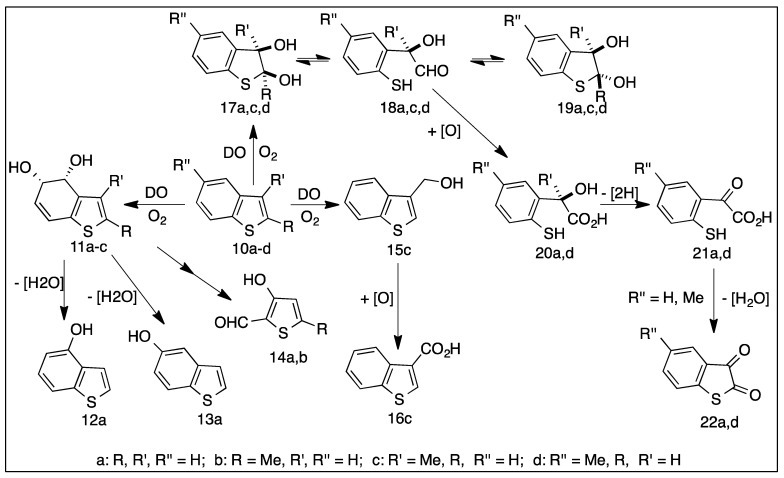
Metabolic pathways of benzo[*b*]thiophenes **10a–d** using *P. putida* strains.

Biodesulfurization of benzo[*b*]thiophene **10a** and alkyl-substituted benzo[*b*]thiophenes, e.g., compounds **10b–d** (Scheme 4), present in fossil fuels, was extensively investigated utilizing wild-type *Pseudomonas* strains (*c.f.* 1. Introduction) [15,16,17,18,19,20,21,22,23,24,25]. Metabolites identified during these studies included: (i) benzo[*b*]thiophene sulfoxides, derived sulfones and dimers, (ii) hydroxybenzo[*b*]thiophenes and dione metabolites, (iii) ring-opened bioproducts (Scheme 4). It was proposed that ring-hydroxylating dioxygenase enzymes (DO), present in wild-type strains of *P. putida*, were responsible for the initial steps in the metabolism of benzo[*b*]thiophene **10a**, leading to *cis*-dihydrodiols, sulfoxides, and sulfoxide dimers [26,27]. Other enzymes, including *cis*-diol dehydrogenases, *cis/trans* isomerases, and catechol dioxygenases, catalyzed further metabolism, to yield catechols, diones, and ring-opened metabolites (Scheme 4). Cytochrome P450 and styrene monooxygenases also catalyzed sulfoxidation of benzo[*b*]thiophenes and benzo[*b*]thiophene-containing drugs (Section 6.3). 

### 4.1. Biotransformations of Benzo[b]thiophenes ***10a***–***d*** to Yield cis-Dihydrodiols ***11a***–***c***

**Scheme 5:**
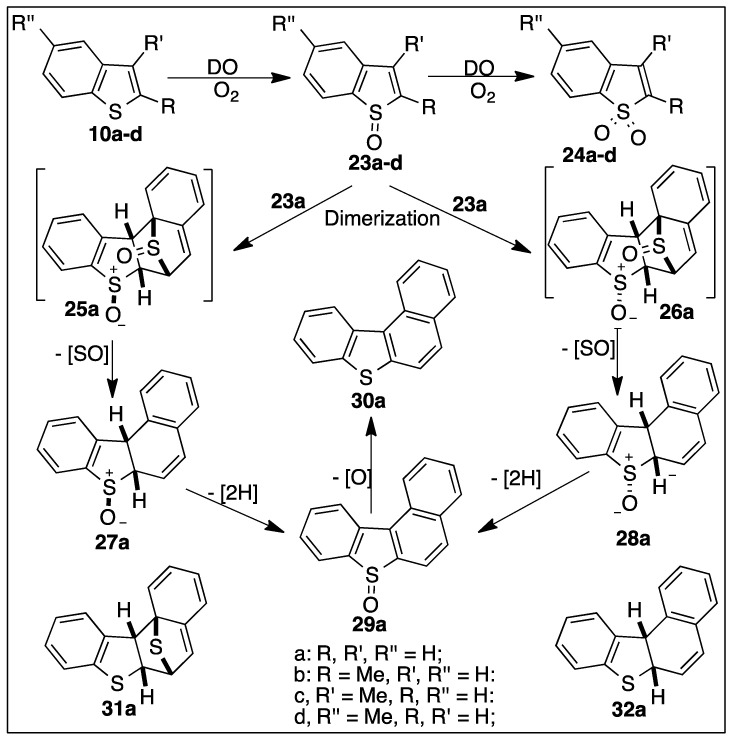
Proposed DO-catalyzed metabolic pathways of benzo[*b*]thiophenes **10a–d**.

Similar bacterial strains used for thiophene substrates **1a–k,** expressing TDO or NDO, were again utilized for benzo[*b*]thiophenes **10a–d** (Scheme 4 and Scheme 5), under similar biotransformation conditions. Toluene dioxygenase (*P. putida* UV4) and isopropylbenzene dioxygenase (*P. putida* RE213) oxidation studies of benzothiophene **10a** indicated that 4*R*,5*S*-dihydrodiol **11a** (>98% *ee*) was a relatively unstable minor product; partial dehydration occurred during the biotransformation, giving hydroxybenzo[*b*]thiophenes (**12a** and **13a**, Scheme 4) [26,81,82,83]. 

TDO-catalyzed *cis*-dihydroxylation of benzo[*b*]thiophene **10b** and **10c** similarly gave *cis*-dihydrodiols 4*R*,5*S*-**11b** (>98% *ee*) and 4*R*,5*S*-**11c** (>98% *ee*), but the presence of a methyl group at the C-5 position inhibited *cis*-dihydroxylation at the 4,5-bond of substrate **10d** (Table 3).

Using NDO-expressing *P. putida* strains (9816/11, NCIMB 8859, and RE204) a much wider range of metabolites from benzo[*b*]thiophenes **10a**-**c** were obtained. Included were alcohol **15c** [83], aldehydes **14a** [26], **14b** [83], carboxylic acid **16c** [83], and *cis*-dihydrodiol **11b [81]**. 

### 4.2. Biotransformations of Benzo[b]thiophenes ***10a***–***d*** to Yield cis-Dihydrodiols ***17a***–***d*** and *trans*-Dihydrodiols ***19a***, ***19d***

As found in TDO-catalyzed biotransformations (*P. putida* UV4) of monocyclic thiophenes **1h–k**, metabolism of benzo[*b*]thiophenes **10a–d** resulted in *cis*-dihydroxylation of the thiophene rings to yield thiohemiacetal *cis*-dihydrodiols **17a, 17b**, and **17d** (Scheme 4). Spontaneous epimerization of *cis* isomers **17a** and **17d,** via undetected aldehyde intermediates **18a** and **18d,** yielded isomeric mixtures with the *trans*-isomers (2*S*,3*R*)-**17a**/(2*R*,3*R*)-**19a** (62% *ee*) and (2*S*,3*R*)-**17d/**(2*R*,3*R*)-**19d** (>98% *ee*, Scheme 4, Table 2) [26,81,82,83]. Tentative evidence for mercaptoaldehyde **18a**, being the intermediate formed during epimerization of diols **17a** and **19a,** was provided by HPLC and UV spectroscopic analyses [27]. 2-Methylbenzo[*b*]thiophene **10b** yielded only *cis*-dihydrodiol (2*S*,3*R*)-**17b** (9% *ee*), which, as a thioacetal, was less susceptible to ring-opening isomerization and isomer **19b** was not found. Dihydrodiols **17c** was obtained as a very minor product but only from NDO-catalyzed oxidation (*P. putida* 9816/11, Table 3).

Isomeric mixtures of benzo[*b*]thiophene dihydrodiol metabolites **17a**/**19a** and **17d/19d** in very polar solvents showed mainly the *trans* isomer (98-100% in CD_3_OD and D_2_O), but, in a less polar solvent, the *cis* isomer was dominant (78-80% in CDCl_3_). Fractional recrystallization of mixtures of dihydrodiol isomers **19a**/**17a** furnished pure samples (>98% *ee*) of isomers **19a** (from MeOH) and **17a** (from CH_2_Cl_2_-hexane) [81,82].

The thiophene ring *cis*-dihydrodiols (**17a,c,d**) and *trans*- dihydrodiol epimers (**19a**, **19d**) are much more stable than the fused benzene ring *cis*-dihydrodiols (**11a**-**c**). This later led to the use of dihydrodiols **17a/19a** in the first synthesis of a thiophene epoxide (Section 5.4) [82].

Benzo[*b*]thiophene 2,3-dione metabolites **22a** and **22d** from substrates **10a** and **10d**, were obtained from dihydrodiol intermediates **17a** and **17d** when using wild-type *P. putida* strains (Scheme 4) [16,20,27]. The metabolic sequence progressed via further intermediates (**18a** and **18d**), → (**20a** and **20d**) → (**21a** and **21d**) and was also observed in other bacterial strains [84]. 

### 4.3. Biotransformations of Benzo[b]thiophenes ***10a***–***d*** to Yield Sulfoxides ***23a***–***d***

The dioxygenase-catalyzed metabolism of the benzo[*b*]thiophene **10a** results in sulfoxidation, dimerization, and formation of tetracyclic thiophenes (Scheme 5).

An authentic sample of the racemic monosulfoxide **23a** was obtained by peroxyacid or dimethyldioxirane oxidation of benzo[*b*]thiophene **10a.** It was found to be sufficiently stable for characterization in solution (CD_3_COCD_3_), but, on concentration, it disproportionated to form benzo[*b*]thiophene **10a** and sulfone **24a** [83]. As expected, the TDO-catalyzed (*P. putida* UV4) sulfoxidation of benzo[*b*]thiophene **10a** yielded the unstable monosulfoxide **23a**. Dimerization then yielded the undetected *bis*-sulfoxides **25a** and **26a**; further metabolism of these intermediates led to the isolation of minor tetracyclic bioproducts **27a–30a** (Scheme 5) [81,83].

Several mechanisms were proposed for the formation of metabolites **27a–30a** (Scheme 5) [19,83,85]. One mechanism involved spontaneous dimerization of monosulfoxide **23a** to form transient *bis*-sulfoxide diastereoisomers **25a** and **26a**, followed by abiotic rearomatization to yield benzo[*b*]naphtho[1,2-*d*]thiophene **30a** [19].

An alternative mechanism proposed dimerization of monosulfoxide **23a**, yielding disulfoxide isomers **25a** and **26a** and extrusion of sulfur monoxide to produce tetracyclic diastereoisomers **27a** and **28a** (Table 3) [81,83]. Thermal extrusion of sulfur monoxide, from thiophene oxide cycloadducts, is a known reaction [86,87]. Direct TDO- and NDO-catalyzed desaturations could account for the formation of benzo[*b*]naphtho[1,2-*d*]thiophene sulfoxide **29a** (3% *ee*, Table 3) from monosulfoxides **27a** and **28a** [88,89]. Reductase-catalyzed deoxygenation of metabolite **29a** would yield benzo[*b*]naphtho[1,2-d]thiophene **30a** [83]. Two additional compounds, **31a** and **32a**, formed when using a *Sphingomonas* strain, were also identified by GC-MS analysis (Scheme 5) [84]. Thermal deoxygenation of sulfoxides **25a–29a**, during analysis, or sulfoxide reductase activity could account for products **31a** and **32a**. The metabolic sequence **10a** → **23a** → **25a, 26a** → **27a, 28a**^.^ → **29a** → **30a** involves several dearomatization and rearomatization steps (Scheme 5).

The advantage of having different types of dioxygenase available is evident from the TDO-catalyzed (*P. putida* UV4) sulfoxidation of 2-methylbenzo[*b*]thiophene **10b** to yield the stable (1*R*)-sulfoxide **23b** (>98% *ee*); NDO-expressing strains (*P. putida* 9816/11 and *E. coli* pF352V) also produced sulfoxide **23b**, but with *P. putida* 8859 an excess (56% *ee*) of the opposite (1*S*) enantiomer was found (Table 3) [83]. NDO-catalyzed oxidation of 3-methylbenzo[*b*]thiophene **10c**, using the same strains, produced the stable sulfoxide **23c** with *P. putida* 8859 yielding an excess of the (1*R*) enantiomer (41% *ee*) [83]. Absolute configurations of sulfoxides **23b** and **23c** were determined by ECD spectroscopy and *ee* values of all sulfoxides by chiral stationary phase HPLC analysis [83].

*Ab initio* computational and quantum chemistry studies predicted that enantiomers of benzo[*b*]thiophene sulfoxide **23a** would undergo pyramidal inversion and racemization at ambient temperature with predicted inversion barriers of (ΔG^≠^ = 22.9-23.9 kcal mol^−1^) [64,65]. Experimental validation was finally provided by the isolation and observed racemization of the (1*R*)-benzo[*b*]thiophene sulfoxide **23a** obtained by a styrene monoxygenase-catalyzed sulfoxidation of substrate **10a** (Section 6.3). Further confirmation of predicted inversion barriers was provided by racemization of (1*R*)-enantiomers of sulfoxides **23b** and **23c,** at ambient temperature. This occurred at a slower rate over a 24h period, and kinetic studies provided pyramidal inversion barriers(ΔG^≠^) of 25.1 and 26.4 kcal mol^−1^, respectively, [83]. Similar inversion barriers are expected for sulfoxide metabolites formed from CYP450-catalyzed sulfoxidation of benzo[*b*]thiophene-containing drugs, e.g., zileuton **10e** and brexpiprazole **10g** (Section 6).

## 5. Dioxygenase-Catalyzed Dearomatization of Tri- and Tetra-Cyclic Thiaarenes

**Figure 4:**
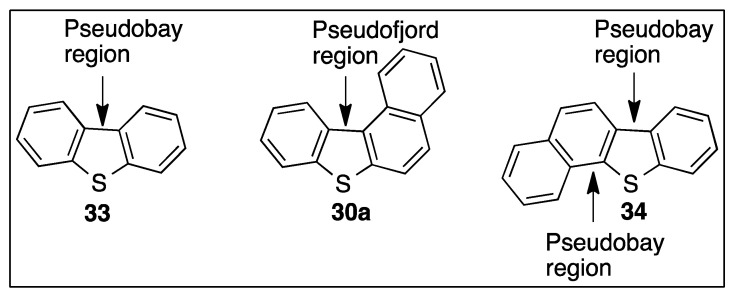
Identification of pseudo-bay and pseudo-fjord regions of polycyclic thiophenes.

Larger thiaarenes, e.g., tricyclic (**33**) and tetracyclic (**30a, 34)** compounds (Figure 4), detected in crude oil, shale, coal, derived tars, and creosote, are generally more resistant to bacterial biodegradation compared with monocyclic and bicyclic thiophenes [15,16,17,18,19,20,21,22,23,24,25]. As observed during dioxygenase-catalyzed *cis*-dihydroxylation of polycyclic arenes, e.g., anthracene or phenanthrene [45,46], linear polycyclic thiophenes appear to be more recalcitrant than angular isomers [24].

The angular junction, between three fused benzene rings found in phenanthrene or chrysene, is classified as a bay region. A strong preference for BPDO-catalyzed *cis*-dihydroxylation of bonds, proximate to a bay region, was found for tricyclic arenes, e.g., phenanthrene [45,46,90] and tetracyclic arenes, e.g., benz[*a*]anthracene or chrysene [91,92,93]. An angular region, between benzene rings fused with a thiophene ring, as in dibenzo[*b,d*]thiophene **33**, or between a fused benzene, thiophene, and naphthalene ring, as in benzo[*b*]naphtho[2,1-*d*]thiophene **34**, is designated a pseudo-bay region (Figure 4).

The angular junction between four fused benzene rings in benzo[*c*]phenanthrene is classified as a fjord region, and BPDO-catalyzed *cis*-dihydroxylation occurred, exclusively, at this region [94]. The region between fused benzene, thiophene, and naphthalene rings, found in benzo[*b*]naphtho[1,2-*d*]thiophene **30a**, is assigned as a pseudo-fjord region.

Ring-hydroxylating dioxygenases with smaller active sites, e.g., TDO, normally only catalyze *cis*-dihydroxylation of mono- and bi-cyclic arenes [4,5,6,7,8,9]. The inability of its active site to accommodate many larger polycyclic thiophenes, e.g., tetracyclic substrates **30a** and **34**, allied to their limited aqueous solubility, are major factors in their resistance to metabolism. The larger active sites of NDO and BPDO are of similar structure (44% sequence identity) [95,96], and the wider entrance to the BPDO active site makes it is among the most successful dioxygenases for binding and biodegrading tri-, tetra-, and penta-cyclic arenes and thiaarenes.

### 5.1. Dioxygenase-Catalyzed Biotransformations of Dibenzo[b,d]thiophenes ***33*** and ***38***

**Scheme 6:**
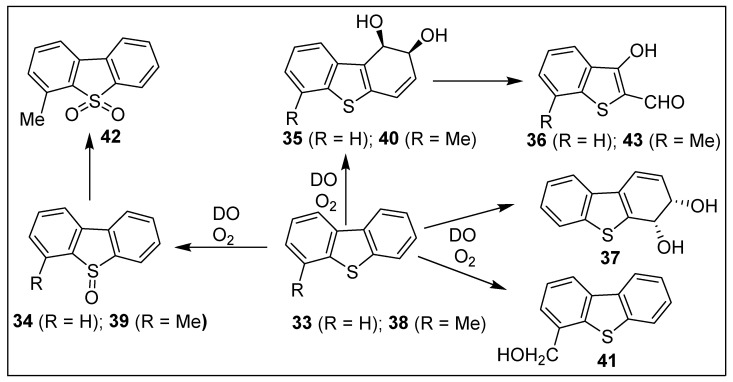
Dioxygenase-catalyzed oxidation of dibenzo[*b,d*]thiophene **33** and 4-methyldibenzo[*b*,*d*]thiophene **38**.

Dibenzo[*b,d*]thiophene **33**, one of the most common polycyclic thiophenes present in crude oil and other fossil fuels, was used as a model substrate for many biodegradation and biodesulfurization studies [97]. Dioxygenase-catalyzed biotransformations (*P. putida, P. jianii, P. alcaligenes, P. stutzeri*, *Burkholdia*, and *Sphingomonas strains*) of substrate **33** yielded sulfoxide **34**. Ring-opened bioproducts like aldehyde 3**6** were derived from *cis*-dihydrodiol intermediate **35** (Scheme 6) [98,99]. NDO-catalyzed sulfoxidation of substrate **33** was also observed using the purified enzyme [100].

Mutant (*P. putida* (9816/11) strain and a recombinant [*E. coli* JM109(DE3)(pDTG141)] strain expressing NDO [100], and mutant (*Beijerinckia*, B8/36) strain expressing BPDO [101], reclassified as *Sphingomonas yanoikuyae* B8/36 [102], were each utilized in the biotransformation of dibenzo[*b,d*]thiophene **33**. In all cases, *cis*-dihydroxylation occurred at the favored pseudo-bay region to yield metabolite (1*R*,2*S*)-1,2-dihydrodiol **35** (>98% *ee*). The minor *cis*-3,4-dihydrodiol **37** was identified from NMR analysis of the unseparated isomeric mixture of *cis*-diols **35**/**37**; it was also detected using other strains (*P. putida* 9816-11, *P. fluorescens* TTC1, and *E. coli* JM109(DE3)(pDTG141) (Scheme 6) [100,103].

Autodock Vina molecular docking studies of dibenzo[*b*,*d*]thiophene **33** as substrate led to the unexpected prediction that it could accommodate a tricyclic substrate within the relatively small active site of TDO [79]. Furthermore, it was predicted that the preferred in vitro orientation of dibenzo[*b,d*]thiophene **33** would result in the formation of (1*R*,2*S*)-dihydrodiol **35**. This proposition was validated in vivo, when TDO-catalyzed (*P. putida* UV4) *cis*-dihydroxylation occurred within the pseudo-bay region, to give exclusively (1*R*,2*S*)-dihydrodiol **35** (>98% *ee*, 16% isolated yield) [79].

Dioxygenase-catalyzed oxidation of 4-methyldibenzo[*b,d*]thiophene **38**, using wild- type *Pseudomonas* strains, yielded a wider range of metabolites including sulfoxide **39,** benzylic alcohol **41**, *cis*-dihydrodiol **40**, sulfone **42**, and aldehyde **43** (Scheme 6) [104]. A kinetic study of the racemization (130 °C) of an enantioenriched sample of sulfoxide **39** produced a higher inversion barrier (ΔG^≠^ = 33.0 kcal mol^−1^) compared with benzo[*b*]thiophene sulfoxides **23a** and **23b**; it was, however, very similar to the calculated value for sulfoxide **34**, ΔG^≠^ = 32.3 kcal mol^−1^ [64].

### 5.2. Dioxygenase-Catalyzed Biotransformation of Benzo[b]naphtho[1,2-d]thiophene ***30a*** and Tetrahydrobenzo[b]naphtho[1,2-d]thiophene ***45***

**Scheme 7:**
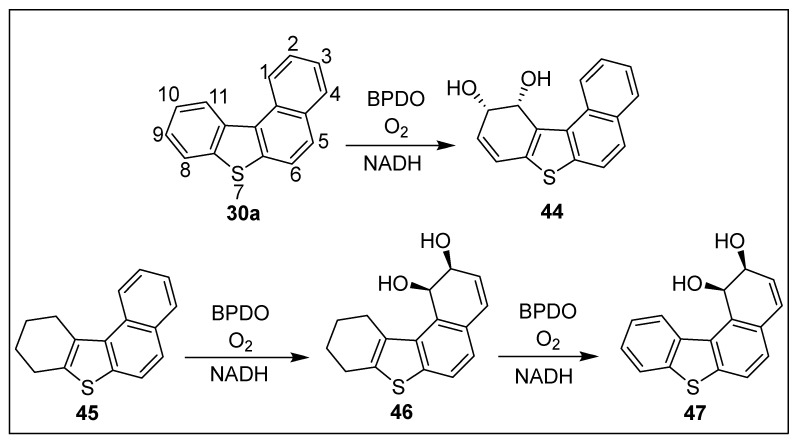
BPDO-catalyzed oxidation of benzo[*b*]naphtho[1,2-*d*]thiophene **30a** and tetrahydrobenzo[*b*]naphtho[1,2-*d*]thiophene **45**.

Benzo[*b*]naphtho[1,2-*d*]thiophene **30a** and the corresponding sulfoxide **29a** were isolated as metabolites from the biotransformations (*P. putida* strains, expressing TDO and NDO, and *Sphingomonas* XLDN2-5) of benzo[*b*]thiophene **10a** (Scheme 5) [12,83,84,97]. No further TDO-catalyzed metabolites of compound **30a** were detected due to its relatively large size. The larger active site of the BPDO (*S. yanoikuyae* B8/36) was able to accommodate and facilitate the metabolism of tetracyclic substrates **30a** and **45** (Scheme 7). BPDO-catalyzed *cis*-dihydroxylation occurred exclusively within the fjord region of benzo[*c*]phenanthrene [94]. Under similar conditions, biotransformation of benzo[*b*]naphtho[1,2-*d*]thiophene **30a** resulted in *cis*-dihydroxylation within the pseudo- fjord region to give (10*S*,11*R*)-dihydrodiol **44** exclusively (>98% *ee*; Scheme 7) [94].

Biotransformation (BPDO) of tetrahydrobenzo[*b*]naphtho[1,2-*d*]thiophene **45** resulted in an unexpected (2:1) mixture of (1*R*,2*S*)- *cis*-dihydrodiols **46** and **47** and was separated by PLC (Scheme 7) [94]. Dioxygenase-catalyzed desaturations (TDO, NDO, or BPDO) are well documented reactions [105,106,107]. A BPDO-catalyzed stepwise double desaturation of metabolite **46**, via a transient dihydrobenzo[*b*]naphtho[1,2-*d*]thiophene intermediate, could account for the minor (10*S*,11*R*) *cis*-dihydrodiol **47**. This study demonstrates the importance of a pseudo-fjord region on the regiochemistry of BPDO-catalyzed *cis*-dihydroxylations.

### 5.3. Dioxygenase-Catalyzed Biotransformation of Benzo[b]naphtho[2,1-d]thiophene ***34***

**Scheme 8:**
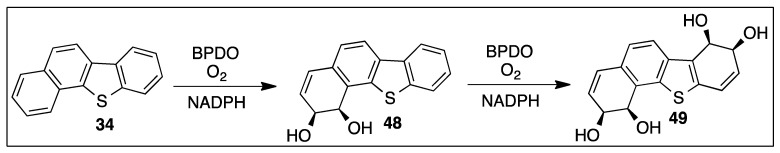
BPDO-catalyzed oxidation of benzo[*b*]naphtho[2,1-*d*]thiophene **34** to yield *cis*-dihydrodiol **48** and *bis*-*cis*-tetrahydrodiol **49**.

Benzo[*b*]naphtho[2,1-*d*]thiophene **34** contains two pseudo-bay regions, and BPDO-catalyzed *cis*-dihydroxylation (*S. yanoikuyae* B8/36) occurred exclusively at one pseudo-bay region to form (1*R*,2*S*)-dihydrodiol **48** as the major metabolite (Scheme 8) [93].

A more polar minor metabolite, resulting from a further *cis*-dihydroxylation within the alternative pseudo-bay region, was identified as *bis*-*cis*-(1*R*,2*S*,7*R*,8*S*)-tetrahydrodiol **49**. Confirmation for this metabolic sequence (Scheme 8) was obtained by using *cis*-dihydrodiol metabolite **48** as a substrate, and, further, *cis*-dihydroxylation yielded *cis*-tetraol **49**. Metabolite **49** was the first identified member of the heteroarene *bis*-*cis*-dihydrodiol series but a similar type of BPDO-catalyzed tetrahydroxylation was observed when using chrysene as a substrate, which contained two bay regions [93].

Benzo[*b*]naphtho[2,1-*d*]thiophene **34** is a carcinogenic polycyclic thiaarene that has been identified as an environmental hazard [13]. It was tested as a substrate for CYP450 monooxygenase-catalyzed studies to identify the suspected metabolites responsible for its mutagenicity and carcinogenicity [108,109]. As found in CYP450 studies of monocyclic thiophenes [53,54,55,56], epoxidation, sulfoxidation, and hydroxylation of tetracyclic thiophene **34** was also reported (Scheme 14, Section 6.3) [108,109].

### 5.4. Application of Thiophene cis-Dihydrodiols in Thiophene Epoxide Synthesis

Enantiopure *cis*-dihydrodiols, derived from enzymatic *cis*-dihydroxylation of substituted arene substrates, have been widely used as precursors in the chemoenzymatic synthesis of many chiral natural products and other types of metabolites, including arene oxides and *trans*-dihydrodiols [4,5,6,7,8,9,44,71]. While few *cis*-dihydrodiol metabolites of heteroarene substrates have been utilized in synthesis, a small number of relatively stable bicyclic metabolites, derived from quinoline, 2-chloroquinoline, and 4-chloroquinoline, have proved to be useful chiral synthons. Thus, chemoenzymatic syntheses of chiral ligands [110,111], chiral metal organic frameworks [112], and arene oxide metabolites, resulting from CYP450-catalyzed oxidations [71], were achieved by the application of these metabolites.

The paucity of reports on the synthetic applications of thiophene sulfoxides and *cis*-dihydrodiols is possibly due to their unavailability and perceived instability. Monocyclic thiophene sulfoxides, although generally considered as unstable, can be stabilized by the presence of bulky substituents. More stable sulfoxides of this type have been used to determine sulfoxide inversion barriers by spectroscopic methods [68,69].

A further example of the synthetic application of thiophene sulfoxides is provided by the reaction of the relatively stable 3,4-di-*tert*-butylthiophene-1-oxide with dimethylacetylene dicarboxylate. This formed an unstable cycloadduct that decomposed spontaneously, providing a source of singlet sulfur monoxide [113]; its reaction with dienes and alkynes delivered a useful synthetic route to thiirane and thiirene oxides.

To test the potential application of thiophene *cis*-dihydrodiols in chemoenzymatic synthesis, it was important to select a relatively stable metabolite. The stability of *cis*- and *trans*-dihydrodiol metabolites **17a** and **19a**, formed by dioxygenase-catalyzed *cis*-dihydroxylation of benzo[*b*]thiophene **10a** (Scheme 4), prompted a study of their use in the synthesis of a thiophene epoxide.

**Scheme 9:**
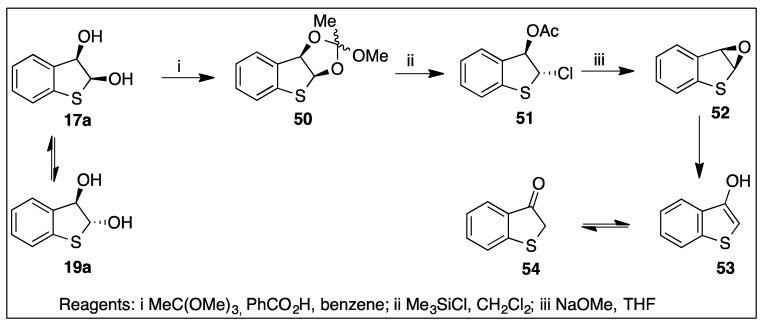
Synthesis of benzo[*b*]thiophene epoxide **52** from *cis*-diol metabolite **17a**.

The three-step reaction sequence in Scheme 9 started from an isomeric mixture of metabolites **17a** and **19a**, proceeded via dioxoles **50**, and *trans*-chloroacetate **51** as intermediates and yielded benzo[*b*]thiophene-2,3-oxide **52**. A similar sequence was used in the synthesis of K-region arene oxides [114]. Epoxide **52** was identified by NMR spectroscopy (THF-d_8_) and MS analysis. On attempted chromatographic purification, it isomerized to a mixture of 3-hydroxybenzo[*b*]thiophene **53** and the keto tautomer **54 [82]**.

## 6. Monooxygenase-Catalyzed Sulfoxidation and Epoxidation of Thiophenes

### 6.1. CYP450-Catalyzed Sulfoxidation of Monocyclic Thiophenes

**Figure 5:**
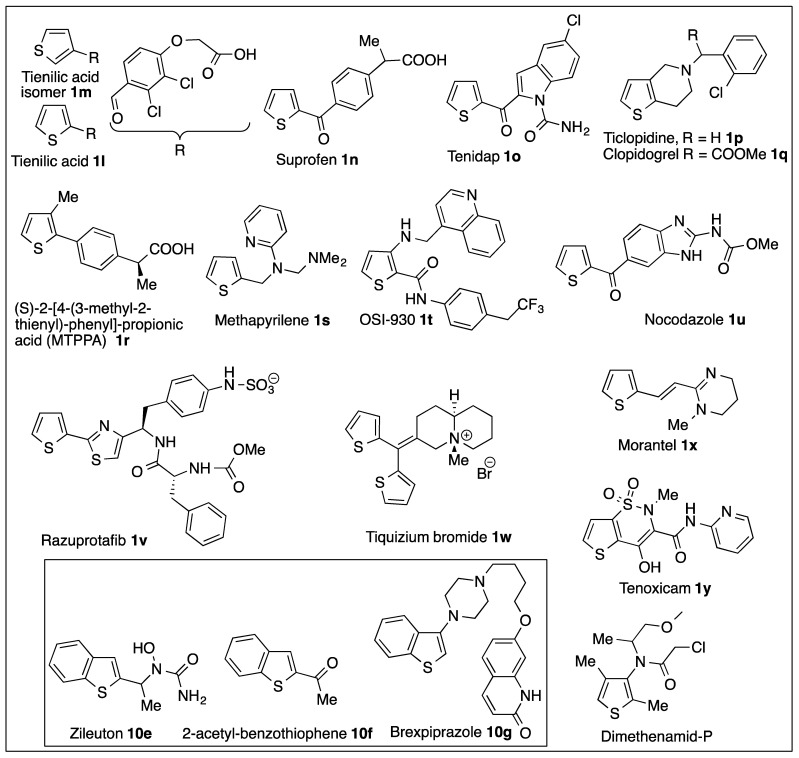
Thiophene- and benzothiophene-containing drugs and analogs tested in metabolic studies.

While thiophenes are considered as contaminants of fossil fuels [14,15,16,17,18,19,20,21,22,23,24,25], the thiophene ring is regarded as a useful bioisosteric building block for many important drugs (Figure 5) [57]. The results from monooxygenase (CYP450)-catalyzed sulfoxidation of thiophenes, in the context of metabolite identification, toxicity, and mechanism of drug metabolism, are rarely compared with sulfoxidations obtained using ring-hydroxylating dioxygenases.

Thiophenes were reported to be hepato- and nephro-toxic to rats [115,116], and these early reports proposed the formation of reactive intermediates, derived from thiophenes rings, which react with cellular nucleophiles; a thiophene sulfoxide *N*-acetylcysteine conjugate was isolated from urine of rats treated with thiophene [117].

In common with the dioxygenase-catalyzed results (Scheme 2), mammalian monooxygenase (CYP450)-catalyzed sulfoxidations of: (i) the model substrates, thiophene **1a**, 2-phenyl-thiophene **1g**, and 3-phenyl thiophene **1k** and (ii) the thiophene-containing drugs analog, tienilic acid isomer **1m** (Figure 5), was observed.

**Scheme 10:**
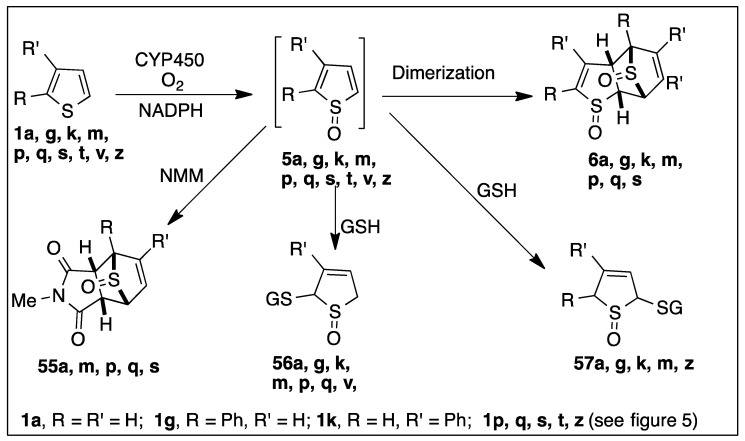
CYP450-catalyzed sulfoxidation of thiophenes **1a**, **g, k, m, p, q, s, t, v, z**.

CYP450 isozymes in liver microsomes, and in recombinant pure form, catalyzed formation of the corresponding unstable sulfoxides as initial metabolites **5a**,**g**,**k,m** (Scheme 10) [52,53,54,55,56,57]. TDO-catalyzed oxidations of thiophenes **1a**,**b**,**g**,**k** also formed sulfoxides **5a**,**b,g**,**k**. In both mono- and di-oxygenase studies, rapid dimerization occurred to give the corresponding dimers **6a,b**,**g**,**k**, respectively (Scheme 2 and Scheme 10). Monocyclic thiophene sulfoxide metabolites **5a,m,p,q, s** were also trapped as cycloadducts by other dienophiles, including *N*-methyl-maleimide (NMM), yielding cycloadducts **55a,m,p,q,s** (Scheme 10) [53,118,119,120]. Michael addition of thiols, e.g., glutathione (GSH), *N*-acetylcysteine, or mercaptoethanol, proved to be a particularly useful method for trapping transient monocyclic sulfoxide metabolites **5a**,**g**,**k**,**m** as adducts **56a**,**g**,**k**,**m** and **57a**,**g**,**k**,**m** (Scheme 10) [52,53,54,55,56,57,58,64,65]. A major advantage of this trapping method, for unstable thiophene sulfoxides, is that these thiols can also trap transient thiophene epoxide metabolites. If the rate of thiol addition is much faster than the rate of thiophene sulfoxide racemization [64,65,66], the configurationally stable thiol adducts could, in principle, provide indirect evidence of sulfoxide enantiomeric excess values.

Cytochrome P450-catalyzed oxidation of the monosubstituted thiophene-containing drugs, tienilic acid isomer **1m**, methapyrilene **1s**, and the disubstituted thiophenes ticlopidine **1p**, clopidogrel **1q**, and OSI-930 **1t** was found to give transient sulfoxide metabolites **5m**,**s**,**p**,**q**, and **5t**, respectively, that rapidly dimerized to **6m**,**s**,**p**,**q,t** (Scheme 10 and Figure 5) [57,121,122]. In the presence of NMM in the incubation, cyclo-adducts **55m**,**p**,**q**, and **55s** were obtained at the cost of the thiophene sulfoxide dimers [120]. Incubation of tri-substituted thiophene dimethenamid-P (Figure 5) herbicide with basidiomycete *Irpex consors* produced a stable thiophene sulfoxide and two isomeric 2-thiolenones [123].

### 6.2. CYP450-Catalyzed Epoxidation of Monocyclic Thiophenes

Both benzene oxide and benzene *cis*-dihydrodiol metabolites have been chemically synthesized and characterized. Scheme 1a illustrates that CYP450-catalyzed epoxidation of mono- and poly-cyclic arene substrates [124,125] can provide an alternative oxidative dearomatization pathway compared to dioxygenase-catalyzed *cis*-hydroxylation. Three major enzymatic oxidative dearomatization pathways (sulfoxidation, *cis*-dihydroxylation, and epoxidation) are possible for mono- and poly-cyclic thiophene substrates (Scheme 1b).

An important difference between *cis*-dihydroxylation and epoxidation of thiophenes is that thiophene *cis*-dihydrodiols have been isolated and fully characterized while, to date, thiophene epoxide metabolites have not. It is important to consider what evidence is available for the involvement of transient benzene oxide metabolites prior to their detection, isolation, and synthesis when attempting to identify transient thiophene epoxide metabolites.

**Scheme 11:**
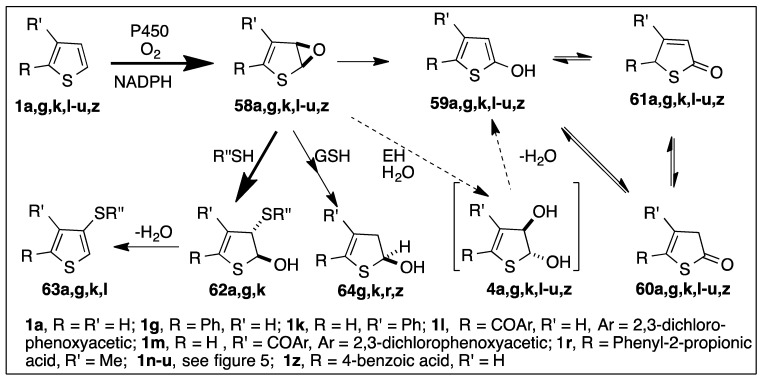
CYP450-catalyzed oxidation of thiophenes **1a**,**g**,**k**,**l–u,z**.

Benzene oxides can: (i) aromatize to phenols, (ii) react with thiols and to yield *trans*-hydroxysulfides, and (iii) hydrolyze to yield *trans*-dihydrodiols, via epoxide hydrolase catalysis. Similarly, thiophene epoxide metabolites derived from thiophenes **1a**,**g**,**k**,**l**, might be expected to aromatize to hydroxythiophenes [122], to form *trans*-hydroxysulfide adducts with thiols, and to hydrolyze, giving *trans*-dihydrodiols as shown in Scheme 11.

The isolation of hydroxythiophene metabolites from monooxygenase-catalyzed model thiophene substrates and thiophene-containing drugs is consistent with thiophene epoxidation and isomerization [126,127]. Thus it was proposed that CYP450-catalyzed oxidation of monosubstituted thiophenes **1a**,**g**,**k** and tienilic acid **1l** could yield unstable thiophene epoxide intermediates **58a**,**g**,**k**,**l** (Scheme 11); spontaneous isomerization of these epoxides could then account for the isolation of monohydroxylated thiophenes **59a**,**g,k**,**l** and their corresponding keto tautomers **60a**,**g**,**k**,**l** and **61a**,**g**,**kl** [54,57,128]. Thus, 2-aroylthiophenic drugs tienilic acid **1l**, suprofen **1n**, tenidap **1o**, and nocodazole **1u** were 5-hydroxylated to produce hydroxythiophene **58l,n,o,u** in equilibrium with the corresponding two thiolactones **60** and **61** having a strong chromophore around 390 nm [126,129,130,131]. Three of these 2-aroylthiophenic drugs were found to exhibit idiosyncratic toxicity and were withdrawn (tienilic acid **1l**, liver toxicity; suprofen **1n**, kidney toxicity), and use of tenidap **1o** was stopped at the end of the clinical studies.

Other thiophene-containing drugs, tiquizium bromide **1w**, morantel **1x**, and tenoxicam **1y**, were also oxidized in microsomal incubations in the thiophene ring, but the position of oxidation was not determined [132,133,134]. For two thiophene-containing drugs, duloxetine and eprosartan, and one benzothiophene-containing drug, raloxifene, metabolic oxidation of the thiophene ring was researched and not detected, other parts of the molecules being oxidized [57].

Following the report of an unusual rearrangement reaction of arenes during enzymatic aromatic hydroxylation, the NIH Shift, this observation was widely used as strong evidence for the intermediacy of transient arene oxides [125,135]. The NIH Shift requires migration of an atom (e.g., D or T) or group and retention at an adjacent site during aromatic hydroxylation of substituted arenes. A similar migration and retention of label was observed during TDO-catalyzed arene *cis*-dihydroxylation and dehydration of the D-labelled *cis*-dihydrodiol metabolites to give phenols [136]. In addition to these two mechanisms for the NIH Shift, other mechanisms have since been reported [137]. The NIH Shift was, however, not observed during CYP450-catalyzed oxidation of thiophenes **1a**,**g**,**k**,**l** during formation of the corresponding hydroxythiophene metabolites **59a**,**g**,**k**,**l** and, therefore, could not be used as evidence for epoxidation.

Arenes can be oxidized by dioxygen and CYP450s forming arene oxides that are then enzymatically hydrolyzed by microsomal epoxide hydrolase (mEH) to give stable *trans*-dihydrodiols. Arene oxides are relatively stable and have been produced by either chemical or enzymatic synthesis. Thiophene *trans*-dihydrodiols **4a,h–k** have been isolated from TDO-catalyzed oxidations of thiophenes as epimers of the initially formed *cis*-dihydrodiols **2a,h–k** [12]; they were relatively stable and did not readily dehydrate. Thus, it was expected that *trans*-dihydrodiols could be formed from thiophene epoxides **58a,g,k,l**, during liver microsomal incubations, as presented in Scheme 11. Neither *trans*-dihydrodiols **4a,g,k,l**, nor the corresponding *cis* epimers **2a,g,k,l**, have been detected as metabolites during CYP450-catalyzed oxidation of any monocyclic thiophenes including compounds **1a,g,k,l** [54,57,128].

Why were thiophene *trans*-dihydrodiols not found? One possibility would be that they are formed below the level of detection. Quantum chemical analysis results predicted that the energy barrier to direct hydrolysis of a thiophene epoxide by water was too high [122]. However, since arene oxide hydrolysis requires catalysis by mEH, a similar outcome might be expected for thiophene epoxides. The size of the active site of mEH is able to accommodate arene oxide sizes from one aromatic ring up to five fused rings and aliphatic epoxides and, thus, should accept small thiophene epoxides. The hydrophobicity of thiophene epoxides should be close to that of arene oxides of similar size. Maybe mEH is not the appropriate enzyme or the half-life of thiophene epoxides is too short for being transferred to the catalytic site of mEH. Despite some thiophenes and thiophene-containing drugs having similar types of substituents, no *trans*-dihydrodiols nor their *cis* epimers have been yet detected directly during microsomal incubations.

The stability of thiophene epoxides [122] could be increased using the same approach adopted for arene oxides [135] that were stabilized by the presence of bulky or electron-withdrawing substituents [137,138]. Despite some model thiophenes and thiophene-containing drugs having similar types of substituents, no epoxide metabolites have been detected directly.

The most convincing indirect evidence for thiophene epoxide intermediates being formed by CYP450-catalyzed oxidation of thiophenes comes from the formation of *trans*-hydroxysulfide adducts (Scheme 11). Nucleophilic attack of thiols, e.g., glutathione or mercaptoethanol, on thiophene epoxide metabolites, e.g., **58a**,**g**,**k,** was found using liver microsomes and individual monooxygenase-expressing strains, e.g., recombinant CYP450 1A [55,56,57,121,128]. The initially formed *trans*-hydroxysulfides, e.g., **62a**,**g**,**k,** were found to epimerize with the corresponding *cis* isomers. Dehydration of *trans*-hydroxysulfides **62a**,**g**,**k** under acidic conditions formed alkylthienyl sulfides **63a**,**g**,**k**

A substituted 2-phenyl thiophene, (*S*)-2-[4-(3-methyl-2-thienyl)phenyl]propionic acid **1r** (MTPPA), as an anti-inflammatory agent, was metabolized to the 5-hydroxy-thiophene metabolite **59r** and its thiolenone tautomers **60r** and **61r** in vivo and in vitro by CYP2C9 [139]. Incubation of thiophene **1r** in the presence of glutathione did not yield adducts, but the detectable proportions of thiolenones decreased with increasing production of an unexpected metabolite. It was identified by NMR spectra and mass spectrometry as the thiophene hydrate 5-hydroxy-4,5-dihydro-3-methylthiophene **64r [140]**. While stable at room temperature, metabolite **64r** dehydrated in an acid medium, yielding thiophene **1r**. Under similar conditions, 2-phenylthiophene **1g** and 4-(2-thienyl)-benzoic acid **1z** substrates also formed thiophene hydrates **64g** and **64z,** but the hydration mechanism was unclear [140].

Relatively few examples of hydrate formation during mono- or di-oxygenase-catalyzed oxidations of arenes or heteroarenes are available. Metabolites **64g**, **64r,** and **64z** are rare examples of heteroarene hydrates formed through CYP450-catalyzed epoxidation of the corresponding thiophenes **1g**,**r,** and **1z**. [140]. Monohydroxylation (CYP450) at allylic or benzylic positions of dihydroarenes yielded polycyclic arene hydrates [141]. Dioxygenase-catalyzed *cis*-dihydroxylation of monosubstituted phenols also produced hydrates, and their keto tautomers, as minor metabolites [78]. The formation of thiophene hydrates and phenol hydrates, during oxidative biotransformations of the corresponding phenol and thiophene substrates, could be regarded as dearomatizations. However, in each case, several steps are involved rather than direct hydration reactions.

Monocyclic thiophene epoxides and sulfoxides have also been identified as biologically reactive intermediates, that react in situ with the enzyme responsible for their production. Several thiophene-containing drugs have been identified as mechanism-based inactivators of cytochrome P450s and to react with the apoenzyme [142,143,144,145,146].

**Scheme 12:**
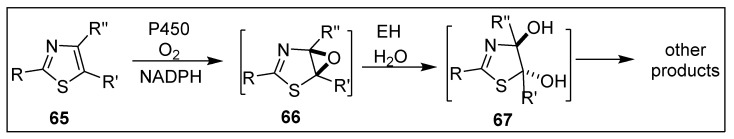
CYP450-catalyzed epoxidation of thiazoles **65**.

No convincing evidence was found for epoxide hydrolase (EH)-catalyzed hydrolysis of thiophene epoxide metabolites, yielding *trans*-dihydrodiols from metabolism of thiophenes and thiophene-containing drugs (Scheme 11). It is, however, noteworthy that the bioactivation of thiazole-containing drugs **65** also involves CYP450-catalyzed formation of unstable thiazole epoxides **66** but is followed by hydrolysis yielding *trans*-dihydrodiols **67** during metabolism (Scheme 12) [147,148,149].

### 6.3. Monooxygenase-Catalyzed Oxidation of Polycyclic Thiophenes

**Scheme 13:**
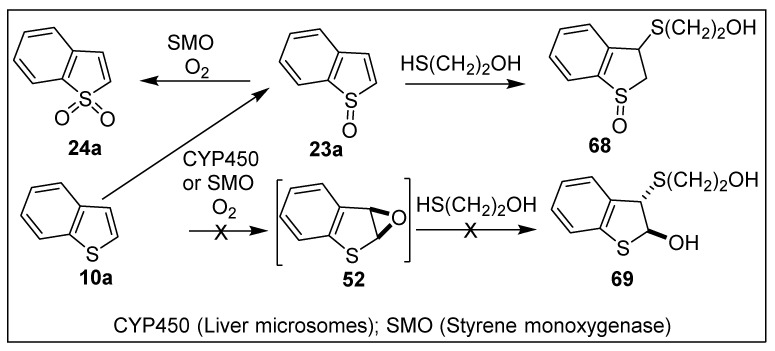
Monooxygenase-catalyzed oxidation of benzo[*b*]thiophene 10a.

Benzo[*b*]thiophene sulfoxide **23a**, obtained by the chemical oxidation of benzo[*b*]thiophene **10a** (Section 4.3), and benzo[*b*]thiophene epoxide **52**, synthesized from *cis*-dihydrodiol **17a** (Scheme 9), were unstable in the neat state but were found to be more stable in solution. This prompted a reexamination of the metabolism of benzo[*b*]thiophene **10a** by monooxygenases from rat liver microsomes (expressing CYP450 enzymes) and a bacterial recombinant strain [*E. coli* BL21 (DE3)] (expressing styrene monooxygenase, SMO) [52,83,150,151]. Following CYP450-catalyzed oxidation of benzo[*b*]thiophene **10a**, benzo[*b*]thiophene sulfoxide **23a** was identified as a major metabolite by HPLC analysis. In the presence of mercaptoethanol (pH 8.5), sulfoxide **23a** was trapped as thiol adduct **68**; no evidence of the epoxide intermediate **52** was obtained by trapping hydroxy sulfide **69** (Scheme 13) [52].

The major metabolite, isolated from styrene monooxygenase-catalyzed [*E. coli* B121 (DE3)] sulfoxidation of benzo[*b*]thiophene **10a**, was (1*R*)-benzo[*b*]thiophene-1-oxide **23a**, [150]; benzo[*b*]thiophene-1,1-dioxide **24a** was found as a very minor metabolite. Sulfoxide **23a**, isolated at 0°C and identified by NMR analysis and slowly racemized over several hours in CDCl_3_ solution at ambient temperature, without decomposition [83].

SMO-catalyzed epoxidation of indene and indole was reported to yield the corresponding epoxides. Indene 1,2-oxide was formed using *E. coli* B121 (DE3) [151], while indirect evidence for indole 2,3-oxide was obtained using *P. putida* strains S12 and CA-3 [152]. Therefore, it was anticipated that SMO might catalyze epoxidation of the bioisosteric substrate benzo[*b*]thiophene **10a**. Despite efforts to detect thiophene oxide **52** (Scheme 13) or to trap it as a hydroxysulfide adduct **69**, epoxide **52** remains an undetected potential metabolite.

CYP450-catalyzed oxidation of the benzo[*b*]thiophene-containing drugs zileuton **10e**, its analog 2-acetylbenzothiophene **10f,** and brexpiprazole **10g** (Figure 5) yielded thiophene sulfoxides without any evidence of epoxide metabolites [57,153,154]. Another benzo[*b*]thiophene-containing drug candidate, JNJ-26990990, was also converted to a sulfoxide[155].

**Scheme 14:**
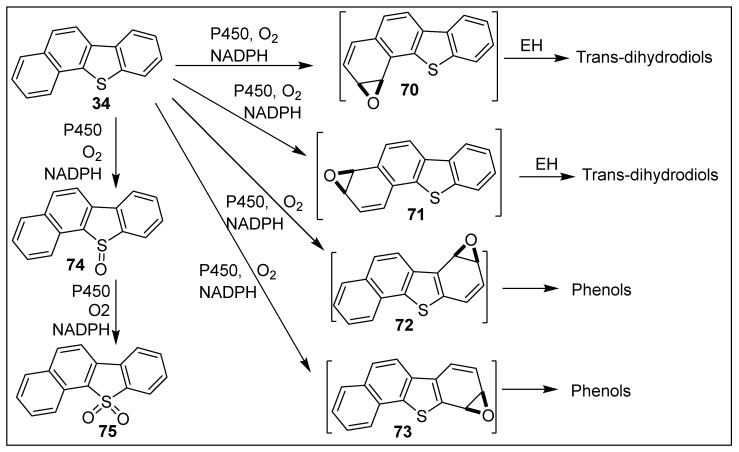
CYP450-catalyzed oxidation of benzo[*b*]naphtho[2,1-*d*]thiophene **34**.

Mono- and di-oxygenase-catalyzed oxidative metabolism of benzo[*b*]naphtho[2,1-*d*]thiophene **34** was studied, due to its mutagenicity, carcinogenicity, and presence in the environment [108,109]. While BPDO-catalyzed oxidation of thiophene **34** yielded *cis*-dihydrodiol **48** and *cis*-tetrahydrodiol **49** (Scheme 8, Section 5.3), an early CYP450-catalyzed metabolism study of substrate **34** yielded only sulfoxide **74** and sulfone **75** (Scheme 14) **[156]**.

Later studies of CYP450-catalyzed metabolism of tetracyclic thiophene **34** also identified sulfoxide **74** and sulfone **75**; in addition, two *trans*-dihydrodiols, derived from the transient arene oxides **70** and **71** and two phenols, resulting from undetected arene oxides **72** and **73,** were identified (Scheme 14) [109]. The proposed CYP450-catalyzed oxidations shown in Scheme 14 involve five enzymatic dearomatization reactions during production of one sulfoxide **74** and four arene oxide intermediates **70–73**.

### 6.4. Monooxygenase-Catalyzed Thiophene Ring Oxidation of Thienopyridine Prodrugs

**Scheme 15:**
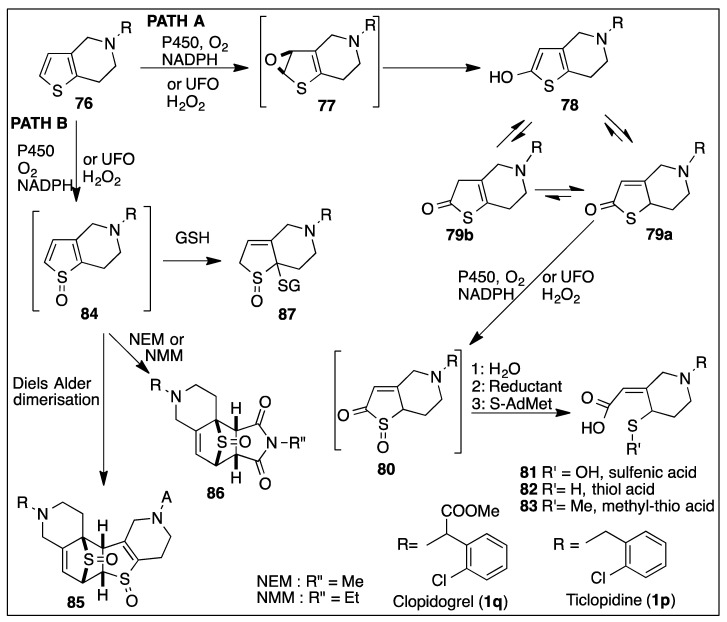
CYP450- or UFO-catalyzed oxidative metabolism of thienopyridine antiplatelet compounds **76** (ticlopidine **1p** and clopidogrel **1q**).

Some important antiplatelet and antiaggregant prodrugs, with a tetrahydro-thienopyridine structure **76** (Ticlopidine **1p**, Clopidogrel **1q**, Figure 5 and Scheme 15), are metabolized in mammals by a series of reactions on the thiophene ring, leading to a thiol acid derivative that inhibits the G-protein ADP receptor P2Y12 [157]. This family of compounds was discovered in 1978 [158], the receptor was located in 2000 and the complex formation of the active metabolite was deciphered during 2009–2013 [158].

The important role played by CYP450 and unspecific fungal peroxygenase (UFO)-catalyzed epoxidation and sulfoxidation is exemplified by the thienopyridine drug metabolism route in Scheme 15. Further reactions of the unstable epoxide and sulfoxide metabolites led to the formation of many other products, including sulfoxide dimers, hydroxythiophenes, thiolactones, sulfenic acids, and thiols.

One of the first steps in the metabolism of thienopyridines **76**, clopidogrel **1q**, and ticlopidine **1p** was oxidation of the thiophene ring by a CYP450 monooxygenase (or UFO peroxygenase) to yield an unstable thiophene epoxide **77**. This intermediate isomerized into a 2-hydroxythiophene **78** and its thiolactone tautomers **79a** and **79b** (Path A, Scheme 15) [159]. Further CYP450- or peroxygenase-catalyzed oxidation of tautomers **78/79a/79b** led to a reactive thiolactone *S*-oxide **80** that hydrolyzed to a sulfenic acid **81** and was trapped using dimedone [120,160,161,162,163,164]. The sulfenic acid metabolite **81** was reduced, in vivo, to thiol acid diastereoisomers **82**, only one being active with the receptor P2Y12. Reducing species, like thiols, ascorbic acid, and tricarboxyethyl-phosphine, were used in vitro [120,160,161,162,163,164,165]. Thiol acid metabolites **82** were eliminated in vivo [166,167] and also as *S*-methylated adducts **83**. The dialkyl sulfides **83** were also obtained in vitro after methylation of **82** by *S*-adenosine-methionine dependent *S*-methyl transferase [168,169].

Some metabolites of the 2-substituted thiophene razuprotafib **1v**, including a methylthioether, may also be formed by Path A with Path B being also involved in formation of other metabolites [170,171]. Metabolic reactions of thiophenes catalyzed by cytochrome P450 were reviewed recently [172].

During the metabolism of ticlopidine **1p** and clopidogrel **1q**, a competitive CYP450 monooxygenation pathway (Path B, Scheme 15) led to thiophene-*S*-oxide **84** and *S*-oxide dimers **85**. The unstable *S*-oxide **84** was trapped by dienophiles, e.g., *N*-methyl- (NMM) and N-ethyl-maleimide (NEM) [119,120] to give product **86**, or thiols, e.g., glutathione (GSH) to give adducts **87** [173]. The oxidation reactions (Path A and Path B) were also catalyzed by unspecific fungal peroxygenases (UFO) in the presence of ascorbic acid and hydrogen peroxide [159].

## 7. Conclusions

A major emphasis of this review has been on the complementary nature of monooxygenase and dioxygenase enzyme activities, in the context of oxidative dearomatization of mono- and poly-cyclic thiophenes. Monooxygenase enzymes, expressed by mammalian, fungal, and bacterial cells, were used in the oxidative aromatization of a wider range of types and sizes of thiophenes compared with ring-hydroxylating dioxygenases.

Wild-type bacteria, expressing ring hydroxylating dioxygenase enzymes, have been employed to metabolize and remove thiophenes from fossil fuels. Mutant and recombinant bacterial strains, expressing these enzymes, were utilized to intercept and scale up the production of thiophene sulfoxide and dihydrodiol metabolites from relatively small thiophene substrates. Factors influencing chemo-, regio-, and stereo-selectivity, stability and mechanisms of dioxygenase-catalyzed oxidations of thiophenes are discussed.

Enantioenriched thiophene sulfoxide metabolites were used to determine inversion barriers and compare with predictions from calculated values. These barriers are of potential importance within the context of individual sulfoxide enantiomers from drugs having different efficacies. A range of inversion barriers were predicted for sulfoxide metabolites obtained during CYP450-catalyzed oxidation of thiophene-containing drugs. The application of a thiophene *cis*-dihydrodiol metabolite in the first synthesis a thiophene epoxide suggests that more stable thiophene epoxides could be obtained using this method.

Lessons can be learned from comparisons of mono- and di-oxygenase-catalyzed oxidation of arenes with thiophenes where metabolite instability is often a major problem. Factors to address this include the use of bulky or electron-withdrawing substituents or benzo-fusion to stabilize thiophene sulfoxides and epoxides. Factors found to influence the thermal stability and dynamic stereochemistry (racemization, *cis*/*trans* isomerization) of metabolites, isolated from dioxygenase-catalyzed oxidation of thiophenes, are also applicable to those derived from monooxygenases. Metabolism of carbocyclic arenes by dioxygenases can yield *cis*-dihydrodiols while transient arene oxides produced by monooxygenases hydrolyze to isolable *trans*-dihydrodiols. Possible reasons are discussed for the almost reverse scenario, where dioxygenases catalyze formation of thiophene *trans*-dihydrodiols as major metablites under aqueous condations while monooxygenases do not.

Further research using suitably substituted thiophene substrates with appropriate dioxygenases could produce *cis*-dihydrodiol metabolites for use in the synthesis of more stable thiophene epoxides and sulfoxides. Monooxygenase-catalyzed production of transient thiophene epoxide metabolites in the presence of a range of epoxide hydrolases might finally lead to *trans*-dihydrodiol production. The question of why model benzo[*b*]thiophenes and benzo[*b*]thiophene-containing drugs and monooxygenases do not appear to produce either epoxides or *trans*-dihydrodiols remains unanswered.

## Figures and Tables

**Table 1:** Relative yields of metabolites from thiophenes **1a**–**g [12]**.

Substrates 1a–g; Strain	5a–g (%)	6a–g (%)	7a–g (%)	2a–g/4a–g (%)	8a–g (%)
**1a, R = R′ = H** (TDO) ^a^	0	79	21	<1 (0.2)	0
**1b, R = Me, R′ = H** (TDO) ^a^	0	25	75	0	0
**1c, R = Et, R′ = H** (TDO) ^a^	0	100	0	0	0
**1d, R = Cl, R′ = H** (TDO) ^a^	0	100	0	0	0
**1e, R = Br, R′ = H** (TDO) ^a^	0	100	0	0	0
**1f, R = I, R′ = H** (TDO) ^a^	0	100	0	0	0
**1g, R = Ph, R′ = H** (TDO) ^a^	0	67	6	0	27
**1g, R = Ph, R′= H** (TDO) ^b^	0	40	0	0	60
**1g, R = Ph, R′ = H** (NDO) ^c^	0	0	0	0	100
**1g, R= Ph, R′ = H** (NDO) ^d^	0	0	0	100	0

^a^ *P. putida* UV4; ^b^ *E. coli* pBAD-Et; ^c^ *E. coli* pDTG141 (NDO); ^d^ *E. coli* pF352V (NDO).

**Table 2:** Relative yields of metabolites from 3-substituted thiophenes **1h**–**k** [12].

Substrates 1h–k; Strain	5h–k (%)	6h–k(%)	7h–k (%)	2h–k/4h–k (%)	9k(%)
**1h**, **R = H, R′ = Me** (TDO) ^a^	0	0	24	76	0
**1i**, **R = H, R′ = Cl** (TDO) ^a^	0	59	0	41	0
**1j**, **R = H, R′ = Br** (TDO) ^a^	0	46	0	54	0
**1k**, **R = H, R′ = Ph** (TDO) ^a^	0	35	0	48	17
**1k**, **R = H, R′ = Ph** (NDO) ^b^	0	2	0	0	98
**1k**, **R = H, R′ = Ph** (NDO) ^c^	0	80	0	20	0

^a^*P. putida* UV4; ^b^ *E. coli* DTG141; ^c^ *E. coli* pF352V.

**Table 3:** Relative yields of metabolites from benzo[*b*]thiophenes **10a–d**.

Substrates 10a–d (Dioxygenase)	11a–c (%)	17a–d (%)	23a–c (%)
**10a**, **R = R′ = R″ = H** (TDO) ^a^	10 ^e^	63	12 ^i^
**10a**, **R = R′ = R″ = H** (NDO) ^b^			100
**10a**, **R = R′ = R″ = H** (NDO) ^c^	0	85	15
**10a**, **R = R′ = R″ = H** (NDO) ^d^	0	86	14
**10b**, **R = Me, R′ = R″ = H** (TDO) ^a^	83	10 ^f^	7
**10b, R = Me, R′ = R″ = H** (NDO) ^b^	0	0 ^g^	90
**10b**, **R = Me, R′ = R″ = H** (NDO) ^c^	90	0	10
**10b**, **R = Me, R′ = R″ = H** (NDO) ^d^	0	60	42
**10c**, **R′ = Me, R = R″ = H** (TDO) ^a^	100	0	0
**10c, R′ = Me, R = R″ = H** (NDO) ^b^	0	0 ^h^	42
**10c**, **R′ = Me, R = R″ = H** (NDO) ^c^	0	10 ^f^	90
**10c**, R**′ = Me, R = R″ = H** (NDO) ^d^	0	0	100
**10d**, **R″ = Me, R = R′ = H** (TDO) ^a^	0	100	0

^a^*P. putida* UV4; ^b^ *P. putida* NCIMB 8859; ^c^ *P. putida* 9816/11; ^d^ *E. coli* pF352V; ^e^ Also (**12a**, 7%), (**13a**, 8%); ^f^ Only *cis*-dihydrodiol **17b** or **17c**; ^g^ Also **14b** (10%); ^h^ Also **15c** (32%), **16c** (26%); ^i^ Also **27a** (4%), **28a** (1%), **29a** (5%), **30a** (2%); (Scheme 5).

## Data Availability

Does not apply.

## Dedication

This paper is dedicated to our mentors, Donald M. Jerina and John W. Daly.

## References

[1] Monks T.J., Butterworth M., Lau S.S. (2010). The fate of benzene-oxide. Chem. Biol. Interact..

[2] Ziffer H., Jerina D.M., Gibson D.T., Kobal V.M. (1973). Absolute stereochemistry of the (+)-cis-1,2-dihydroxy-3-methylcyclohexa-3,5-diene produced from toluene by Pseudomonas putida. J. Am. Chem. Soc..

[3] Heider J., Fuchs G. (1997). Anaerobic Metabolism of Aromatic Compounds. Eur. J. Biochem..

[4] Boyd D.R., Sheldrake G.N. (1998). The dioxygenase-catalysed formation of vicinal cis-diols. Nat. Prod. Rep..

[5] Hudlicky T., Gonzalez D., Gibson D.T. (1999). Enzymatic dihydroxylation of aromatics in enantioselective synthesis: Expanding asymmetric methodology. Aldrichim. Acta.

[6] Johnson R.A. (2004). Microbial Arene Oxidations. Org. React..

[7] Lewis S.E. (2016). Asymmetric Dearomatization Under Enzymatic Conditions. Asymmetric Dearomatization Reactions.

[8] Hudlicky T. (2018). Benefits of Unconventional Methods in the Total Synthesis of Natural Products. ACS Omega.

[9] Taher E.S., Banwell M., Buckler J., Yan Q., Lan P. (2017). The Exploitation of Enzymatically-Derivedcis-1,2-Dihydrocatechols and Related Compounds in the Synthesis of Biologically Active Natural Products. Chem. Rec..

[10] Solà M. (2017). Why Aromaticity Is a Suspicious Concept? Why?. Front. Chem..

[11] Horner K.E., Karadakov P.B. (2013). Chemical Bonding and Aromaticity in Furan, Pyrrole, and Thiophene: A Magnetic Shielding Study. J. Org. Chem..

[12] Boyd D.R., Sharma N.D., Gunaratne N., Haughey S.A., Kennedy M.A., Malone J.F., Allen C.C.R., Dalton H. (2003). Dioxygenase-catalysed oxidation of monosubstituted thiophenes: Sulfoxidation versus dihydrodiol formation. Org. Biomol. Chem..

[13] Jacob J. (1990). Sulfur Analogues of Polycyclic Aromatic Hydrocarbons (Thiaarenes).

[14] Boshagh F., Rahmani M., Rostami K., Yousefifar M. (2021). Key Factors Affecting the Development of Oxidative Desulfurization of Liquid Fuels: A Critical Review. Energy Fuels.

[15] Fedorak P., Orr W., White C.M. (1990). Microbial metabolism of organosulfur compounds in petroleum. Geochemistry of Sulfur in Fossil Fuels.

[16] Fedorak P.M., Grbić-Galić D. (1991). Aerobic Microbial Cometabolism of Benzothiophene and 3-Methylbenzothiophene. Appl. Environ. Microbiol..

[17] Saftic S., Fedorak P.M., Andersson J.T. (1992). Diones, sulfoxides, and sulfones from the aerobic cometabolism of methylbenzothiophenes by Pseudomonas strain BT1. Environ. Sci. Technol..

[18] Fedorak P.M., Peakman T.M. (1992). Aerobic microbial metabolism of some alkylthiophenes found in petroleum. Biodegradation.

[19] Kropp K.G., Gonçalves J.A., Andersson J.T., Fedorak P.M. (1994). Microbially Mediated Formation of Benzonaphthothiophenes from Benzo[ b ]thiophenes. Appl. Environ. Microbiol..

[20] Kropp K.G., Goncalves J.A., Andersson J.T., Fedorak P.M. (1994). Bacterial Transformations of Benzothiophene and Methylbenzothiophenes. Environ. Sci. Technol..

[21] Kropp K.G., Saftic S., Andersson J.T., Fedorak P.M. (1996). Transformations of six isomers of dimethylbenzothiophene by three Pseudomonas strains. Biodegradation.

[22] Fedorak P.M., Coy D.L., Peakman T.M. (1996). Microbial metabolism of some 2,5-substituted thiophenes. Biodegradation.

[23] Kropp K.G., Andersson J.T., Fedorak P.M. (1997). Bacterial transformations of 1,2,3,4-tetrahydrodibenzothiophene and dibenzothiophene. Appl. Environ. Microbiol..

[24] Kropp K.G., Andersson J.T., Fedorak P.M. (1997). Bacterial transformations of naphthothiophenes. Appl. Environ. Microbiol..

[25] Bressler D.C., Norman J.A., Fedorak P.M. (1997). Ring cleavage of sulfur heterocycles: How does it happen?. Biodegradation.

[26] Boyd D.R., Sharma N.D., Boyle R., McMurray B.T., Evans T.A., Malone J.F., Dalton H., Chima J., Sheldrake G.N. (1993). Biotransformation of unsaturated heterocyclic rings by Pseudomonas putida to yield cis-diols. J. Chem. Soc. Chem. Commun..

[27] Eaton R.W., Nitterauer J.D. (1994). Biotransformation of benzothiophene by isopropylbenzene-degrading bacteria. J. Bacteriol..

[28] Lee K., Brand J., Gibson D. (1995). Stereospecific Sulfoxidation by Toluene and Naphthalene Dioxygenases. Biochem. Biophys. Res. Commun..

[29] Bugg T.D., Ramaswamy S. (2008). Non-heme iron-dependent dioxygenases: Unravelling catalytic mechanisms for complex enzymatic oxidations. Curr. Opin. Chem. Biol..

[30] Barry S.M., Challis G.L. (2013). Mechanism and Catalytic Diversity of Rieske Non-Heme Iron-Dependent Oxygenases. ACS Catal..

[31] Ibrahim S.R.M., Abdallah H.M., El-Halawany A.M., Mohamed G.A. (2016). Naturally occurring thiophenes: Isolation, purification, structural elucidation, and evaluation of bioactivities. Phytochem. Rev..

[32] Misawa N., Shindo K., Takahashi H., Suenaga H., Iguchi K., Okazaki H., Harayama S., Furukawa K. (2002). Hydroxylation of various molecules including heterocyclic aromatics using recombinant Escherichia coli cells expressing modified biphenyl dioxygenase genes. Tetrahedron.

[33] Boyd D.R., Sharma N.D., Hand M.V., Groocock M.R., Kerley N.A., Dalton H., Chima J., Sheldrake G.N. (1993). Stereodirecting substituent effects during enzyme-catalysed synthesis of cis-dihydrodiol metabolites of 1,4-disubstituted benzene substrates. J. Chem. Soc. Chem. Commun..

[34] Yildirim S., Franco T.T., Wohlgemuth R., Kohler H.-P.E., Witholt B., Schmid A. (2005). Recombinant Chlorobenzene Dioxygenase fromPseudomonas sp. P51: A Biocatalyst for Regioselective Oxidation of Aromatic Nitriles. Adv. Synth. Catal..

[35] Hoye T.R., Jeffrey C.S., Shao F. (2007). Mosher ester analysis for the determination of absolute configuration of stereogenic (chiral) carbinol carbons. Nat. Protoc..

[36] Boyd D.R., Dorrity M.R.J., Hand M.V., Malone J.F., Sharma N.D., Dalton H., Gray D.J., Sheldrake G.N. (1991). Enantiomeric excess and absolute configuration determination of cis-dihydrodiols from bacterial metabolism of monocyclic arenes. J. Am. Chem. Soc..

[37] Boyd D.R., Sharma N.D., Boyle R., McMordie R.S., Chima J., Dalton H. (1992). A H-nmr method for the determination of enantiomeric excess and absolute configuration of cis-dihydrodiol metabolites of polycyclic arenes and heteroarenes. Tetrahedron Lett..

[38] Burgess K., Porte A.M. (1994). A Reagent for Determining Optical Purities of Diols by Formation of Diastereomeric Arylboronate Esters. Angew. Chem. Int. Ed..

[39] Resnick S.M., Torok D.S., Lee K., Brand J.M., Gibson D.T. (1995). Regiospecific and streoselective hydroxylation pf 1-indanone and 2-indanone by naphthalene dioxygenase and toluene dioxygenase. Appl. Environ. Microbiol..

[40] Boyd D.R., Sharma N.D., Goodrich P.A., Malone J.F., McConville G., Harrison J.S., Stevenson P.J., Allen C.C. (2017). Enantiopurity and absolute configuration determination of arenecis-dihydrodiol metabolites and derivatives using chiral boronic acids. Chirality.

[41] Gawronski J.K., Kwit M., Boyd D.R., Sharma N.D., Malone A.J.F., Drake A.F. (2005). Absolute Configuration, Conformation, and Circular Dichroism of Monocyclic Arene Dihydrodiol Metabolites: It is All Due to the Heteroatom Substituents. J. Am. Chem. Soc..

[42] Kwit M., Gawroński J., Boyd D.R., Sharma N.D., Kaik M., More O’Ferrall R.A., Kudavalli J.S. (2008). Toluene Dioxygenase-Catalyzed Synthesis of cis-Dihydrodiol Metabolites from 2-Substituted Naphthalene Substrates: Assignments of Absolute Configurations and Conformations from Circular Dichroism and Optical Rotation Measurements. Chem.—Eur. J..

[43] Kwit M., Gawronski J., Sbircea L., Sharma N.D., Kaik M., Boyd D.R. (2009). Circular dichroism spectra, optical rotations and absolute configurations ofcis-dihydrodiol metabolites of quinoline and derivatives: The role of the nitrogen atom. Chirality.

[44] Boyd D.R., Sharma N.D., Ljubez V., McGeehin P.K.M., Stevenson P.J., Blain M., Allen C.C.R. (2013). Chemoenzymatic synthesis of monocyclic arene oxides and arene hydrates from substituted benzene substrates. Org. Biomol. Chem..

[45] Jerina D.M., Selander H., Yagi H., Wells M.C., Davey J.F., Mahadevan V., Gibson D.T. (1976). Dihydrodiols from anthracene and phenanthrene. J. Am. Chem. Soc..

[46] Parales R.E., Resnick S.M., Yu C.-L., Boyd D.R., Sharma N.D., Gibson D.T. (2000). Regioselectivity and Enantioselectivity of Naphthalene Dioxygenase during Arene cis -Dihydroxylation: Control by Phenylalanine 352 in the α Subunit. J. Bacteriol..

[47] Boyd D.R., Sharma N.D., Coen G.P., Hempenstall F., Ljubez V., Malone J.F., Allen C.C.R., Hamilton J.T.G. (2008). Regioselectivity and stereoselectivity of dioxygenase catalysed cis-dihydroxylation of mono- and tri-cyclic azaarene substrates. Org. Biomol. Chem..

[48] Alien C.C.R., Boyd D.R., Dalton H., Sharma N.D., Haughey S.A., McMordie R.A.S., McMurray B.T., Sheldrake G.N., Sproule K. (1995). Sulfoxides of high enantiopurity from bacterial dioxygenase-catalysed oxidation. J. Chem. Soc. Chem. Commun..

[49] Boyd D.R., Sharma N.D., Haughey S.A., Kennedy M.A., McMurray B.T., Sheldrake G.N., Allen C.C.R., Dalton H., Sproule K. (1998). Toluene and naphthalene dioxygenase-catalysed sulfoxidation of alkyl aryl sulfides. J. Chem. Soc. Perkin Trans..

[50] Kerridge A., Willetts A., Holland H. (1999). Stereoselective oxidation of sulfides by cloned naphthalene dioxygenase. J. Mol. Catal. B Enzym..

[51] Boyd D.R., Sharma N.D., Byrne B.E., Haughey S.A., Kennedy M.A., Allen C.C.R. (2004). Dioxygenase-catalysed oxidation of alkylaryl sulfides: Sulfoxidation versus cis-dihydrodiol formation. Org. Biomol. Chem..

[52] Mansuy D., Valadon P., Erdelmeier I., Lopez-Garcia P., Amar C., Girault J.P., Dansette P.M. (1991). Thiophene S-oxides as new reactive metabolites: Formation by cytochrome P-450 dependent oxidation and reaction with nucleophiles. J. Am. Chem. Soc..

[53] Treiber A., Dansette P.M., El Amri H., Girault J.-P., Ginderow D., Mornon A.J.-P., Mansuy D. (1997). Chemical and Biological Oxidation of Thiophene: Preparation and Complete Characterization of Thiophene S-Oxide Dimers and Evidence for Thiophene S-Oxide as an Intermediate in Thiophene Metabolism In Vivo and In Vitro. J. Am. Chem. Soc..

[54] Dansette P.M., Bertho G., Mansuy D. (2005). First evidence that cytochrome P450 may catalyze both S-oxidation and epoxidation of thiophene derivatives. Biochem. Biophys. Res. Commun..

[55] Medower C., Wen L., Johnson W.W. (2008). Cytochrome P450 Oxidation of the Thiophene-Containing Anticancer Drug 3-[(Quinolin-4-ylmethyl)-amino]-thiophene-2-carboxylic Acid (4-Trifluoromethoxy-phenyl)-amide to an Electrophilic Intermediate. Chem. Res. Toxicol..

[56] Rademacher P.M., Woods C.M., Huang Q., Szklarz G.D., Nelson S.D. (2012). Differential Oxidation of Two Thiophene-Containing Regioisomers to Reactive Metabolites by Cytochrome P450 2C9. Chem. Res. Toxicol..

[57] Gramec D., Mašič L.P., Dolenc M.S. (2014). Bioactivation Potential of Thiophene-Containing Drugs. Chem. Res. Toxicol..

[58] Heine T., Scholtissek A., Hofmann S., Koch R., Tischler D. (2019). Accessing Enantiopure Epoxides and Sulfoxides: Related Flavin-Dependent Monooxygenases Provide Reversed Enantioselectivity. ChemCatChem.

[59] Yang J., Yuan Z., Zhou Y., Zhao J., Yang M., Cheng X., Ou G., Chen Y. (2016). Asymmetric reductive resolution of racemic sulfoxides by recombinant methionine sulfoxide reductase from a pseudomonas monteilii strain. J. Mol. Catal. B Enzym..

[60] Yang J., Wen Y., Peng L., Chen Y., Cheng X., Chen Y.-Z. (2019). Identification of MsrA homologues for the preparation of (R)-sulfoxides at high substrate concentrations. Org. Biomol. Chem..

[61] Luckarift H.R., Dalton H., Sharma N.D., Boyd D.R., Holt R.A. (2004). Isolation and characterisation of bacterial strains containing enantioselective DMSO reductase activity: Application to the kinetic resolution of racemic sulfoxides. Appl. Microbiol. Biotechnol..

[62] Boyd D.R., Sharma N.D., King A.W.T., Shepherd S.D., Allen C.C.R., Holt R.A., Luckarift H.R., Dalton H. (2004). Stereoselective reductase-catalysed deoxygenation of sulfoxides in aerobic and anaerobic bacteria. Org. Biomol. Chem..

[63] Anselmi S., Aggarwal N., Moody T.S., Castagnolo D. (2020). Unconventional Biocatalytic Approaches to the Synthesis of Chiral Sulfoxides. ChemBioChem.

[64] Verlinden S., Woller T., De Proft F., Verniest G., Alonso M. (2019). Towards the Design of Optically Active Thiophene S-Oxides using Quantum Chemistry. Chem.—Eur. J..

[65] Jenks W.S., Matsunaga N., Gordon M. (1996). Effects of Conjugation and Aromaticity on the Sulfoxide Bond. J. Org. Chem..

[66] Bongini A., Arbizzani C., Barbarella G., Zambianchi M., Mastragostino M. (2000). Thiophene S-oxides: Orbital energies and electrochemical properties. Chem. Commun..

[67] Pouzet P., Erdelmeier I., Ginderow D., Mornon J.-P., Dansette P., Mansuy D. (1995). Thiophene S-oxides: Convenient preparation, first complete structural characterization and unexpected dimerization of one of them, 2,5-diphenylthiophene-1-oxide. J. Chem. Soc. Chem. Commun..

[68] Otani T., Miyoshi M., Shibata T., Matsuo T., Hashizume D., Tamao K. (2017). Thermally Stable Monosubstituted Thiophene 1-Oxide and 1-Imides Stabilized by a Bulky Rind Group at Their 3-Position: Synthesis, Structure, and Inversion Barriers on the Sulfur Atom. Bull. Chem. Soc. Jpn..

[69] Mock W.L. (1970). Stable thiophene sulfoxides. J. Am. Chem. Soc..

[70] Xiong F., Yang B.-B., Zhang J., Li L. (2018). Enantioseparation, Stereochemical Assignment and Chiral Recognition Mechanism of Sulfoxide-Containing Drugs. Molecules.

[71] Boyd D.R., Sharma N.D., Loke P.L., Carroll J.G., Stevenson P.J., Hoering P., Allen C.C.R. (2021). Toluene Dioxygenase-Catalyzed cis-Dihydroxylation of Quinolines: A Molecular Docking Study and Chemoenzymatic Synthesis of Quinoline Arene Oxides. Front. Bioeng. Biotechnol..

[72] Höring P., Rothschild-Mancinelli K., Sharma N.D., Boyd D.R., Allen C.C.R. (2016). Oxidative biotransformations of phenol substrates catalysed by toluene dioxygenase: A molecular docking study. J. Mol. Catal. B Enzym..

[73] Friemann R., Lee K., Brown E., Gibson D.T., Eklund H., Ramaswamy S. (2008). Structures of the multicomponent Rieske non-heme iron toluene 2,3-dioxygenase enzyme system. Acta Crystallogr. Sect. D Biol. Crystallogr..

[74] Karlsson A., Parales J.V., Parales R.E., Gibson D.T., Eklund H., Ramaswamy S. (2003). Crystal Structure of Naphthalene Dioxygenase: Side-on Binding of Dioxygen to Iron. Science.

[75] Vila M.A., Pazos M., Iglesias C., Veiga N., Seoane G., Carrera I. (2016). Toluene Dioxygenase-Catalysed Oxidation of Benzyl Azide to Benzonitrile: Mechanistic Insights for an Unprecedented Enzymatic Transformation. ChemBioChem.

[76] Vila M.A., Umpiérrez D., Seoane G., Rodríguez S., Carrera I., Veiga N. (2016). Computational insights into the oxidation of mono- and 1,4 disubstituted arenes by the Toluene Dioxygenase enzymatic complex. J. Mol. Catal. B Enzym..

[77] Vila M.A., Umpiérrez D., Veiga N., Seoane G., Carrera I., Giordano S.R. (2017). Site-Directed Mutagenesis Studies on the Toluene Dioxygenase Enzymatic System: Role of Phenylalanine 366, Threonine 365 and Isoleucine 324 in the Chemo-, Regio-, and Stereoselectivity. Adv. Synth. Catal..

[78] Boyd D.R., Sharma N.D., McIntyre P.B.A., Stevenson P.J., McRoberts W.C., Gohil A., Hoering P., Allen C. (2017). Enzyme-Catalysed Synthesis of Cyclohex-2-en-1-one *cis*-Diols from Substituted Phenols, Anilines and Derived 4-Hydroxycyclohex-2-en-1-ones. Adv. Synth. Catal..

[79] Boyd D.R., Sharma N.D., Brannigan I.N., McGivern C.J., Nockemann P., Stevenson P.J., McRoberts C., Hoering P., Allen C.C.R. (2019). Cis-Dihydroxylation of Tricyclic Arenes and Heteroarenes Catalyzed by Toluene Dioxygenase: A Molecular Docking Study and Experimental Validation. Adv. Synth. Catal..

[80] González-Rosende M.E., Castillo E., Jennings W.B., Malone J.F. (2017). Stereodynamics and edge-to-face CH–π aromatic interactions in imino compounds containing heterocyclic rings. Org. Biomol. Chem..

[81] Boyd D.R., Sharma N.D., Brannigan I.N., Haughey S.A., Malone J.F., Clarke D.A., Dalton H. (1996). Dioxygenase-catalysed formation of cis/trans-dihydrodiol metabolites of mono- and bi-cyclic heteroarenes. Chem. Commun..

[82] Boyd D.R., Sharma N.D., Brannigan I.N., Evans T.A., Haughey S.A., McMurray B.T., Malone J.F., McIntyre P.B.A., Stevenson P.J., Allen C.C.R. (2012). Toluene dioxygenase-catalyzed cis-dihydroxylation of benzo[b]thiophenes and benzo[b]furans: Synthesis of benzo[b]thiophene 2,3-oxide. Org. Biomol. Chem..

[83] Boyd D.R., Sharma N.D., McMurray B., Haughey S.A., Allen C.C.R., Hamilton J.T.G., McRoberts W.C., More O’Ferrall R.A., Nikodinovic-Runic J., Coulombel L.A. (2011). Bacterial dioxygenase- and monooxygenase-catalysed sulfoxidation of benzo[b]thiophenes. Org. Biomol. Chem..

[84] Gai Z.H., Yu B., Wang X.Y., Deng Z.X., Xu P. (2008). Microbial transformation of benzothiophenes, with carbazole as the auxiliary substrate, by Sphingomonas sp strain XLDN2–5. Microbiology.

[85] Boyd D.R., Sharma N.D., Haughey S.A., Malone J.F., McMurray B.T., Sheldrake G.N., Allen C.C.R., Dalton H. (1996). Enantioselective dioxygenase-catalysed formation and thermal racemisation of chiral thiophene sulfoxides. Chem. Commun..

[86] Thiemann T., Fujii H., Ohira D., Arima K., Li Y., Mataka S. (2003). Cycloaddition of thiophene S-oxides to allenes, alkynes and to benzyne. New J. Chem..

[87] Joost M., Nava M., Transue W.J., Martin-Drumel M.-A., McCarthy M.C., Patterson D., Cummins C.C. (2018). Sulfur monoxide thermal release from an anthracene-based precursor, spectroscopic identification, and transfer reactivity. Proc. Natl. Acad. Sci. USA.

[88] Torok D.S., Resnick S.M., Brand J.M., Cruden D.L., Gibson D.T. (1995). Desaturation and oxygenation of 1,2-dihydronaphthalene by toluene dioxygenase and naphthalene dioxygenase. J. Bacteriol..

[89] Boyd D.R., Sharma N.D., Kerley N.A., McMordie R.A.S., Sheldrake G.N., Williams P., Dalton H. (1996). Dioxygenase-catalysed oxidation of dihydronaphthalenes to yield arene hydrate and cis-dihydro naphthalenediols. J. Chem. Soc. Perkin Trans..

[90] Koreeda M., Akhtar M.N., Boyd D.R., Neill J.D., Gibson D.T., Jerina D.M. (1978). Absolute stereochemistry of cis-1,2-, trans-1,2-, and cis-3,4-dihydrodiol metabolites of phenanthrene. J. Org. Chem..

[91] Jerina D.M., Van Bladeren P.J., Yagi H., Gibson D.T., Mahadevan V., Neese A.S., Koreeda M., Sharma N.D., Boyd D.R. (1984). Synthesis and absolute configuration of the bacterial cis-1,2-, cis-8,9-, and cis-10,11-dihydro diol metabolites of benz[a]anthracene formed by strain of Beijerinckia. J. Org. Chem..

[92] Boyd D.R., Sharma N.D., Agarwal R., Resnick S.M., Schocken M.J., Gibson D.T., Sayer J.M., Yagi H., Jerina D.M. (1997). Bacterial dioxygenase-catalysed dihydroxylation and chemical resolution routes to enantiopure cis-dihydrodiols of chrysene. J. Chem. Soc. Perkin Trans..

[93] Boyd D.R., Sharma N.D., Hempenstall F., Kennedy M.A., Malone J.F., Allen C.C.R., Resnick S.M., Gibson D.T. (1999). bis-cis-Dihydrodiols: A New Class of Metabolites Resulting from Biphenyl Dioxygenase-Catalyzed Sequential Asymmetriccis-Dihydroxylation of Polycyclic Arenes and Heteroarenes. J. Org. Chem..

[94] Boyd D.R., Sharma N.D., Harrison J.S., Kennedy M.A., Allen C.C.R., Gibson D.T. (2001). Regio- and stereo-selective dioxygenase-catalysed cis-dihydroxylation of fjord-region polycyclic arenes. J. Chem. Soc. Perkin Trans..

[95] Ferraro D.J., Brown E.N., Yu C.L., Parales R.E., Gibson D.T., Ramaswamy S. (2007). Structural investigations of the ferredoxin and terminal oxygenase components of the biphenyl 2,3-dioxygenase from Sphingobium yanoikuyae B1. BMC Struct. Biol..

[96] Ferraro D.J., Okerlund A., Brown E., Ramaswamy S. (2017). One enzyme, many reactions: Structural basis for the various reactions catalyzed by naphthalene 1,2-dioxygenase. IUCrJ.

[97] Gai Z., Yu B., Li L., Wang Y., Ma C., Feng J., Deng Z., Xu P. (2007). Cometabolic Degradation of Dibenzofuran and Dibenzothiophene by a Newly Isolated Carbazole-Degrading Sphingomonas sp. Strain. Appl. Environ. Microbiol..

[98] Kodama K., Umehara K., Shimizu K., Nakatani S., Minoda Y., Yamada K. (1973). Identification of Microbial Products from Dibenzothiophene and Its Proposed Oxidation Pathway. Agric. Biol. Chem..

[99] Kodama K., Nakatani S., Umehara K., Shimizu K., Minoda Y., Yamada K. (1970). Microbial Conversion of Petro-sulfur Compounds. Agric. Biol. Chem..

[100] Resnick S.M., Gibson D.T. (1996). Regio- and stereospecific oxidation of fluorene, dibenzofuran, and dibenzothiophene by naphthalene dioxygenase from Pseudomonas sp strain NCIB 9816–4. Appl. Environ. Microbiol..

[101] Laborde A.L., Gibson D.T. (1977). Metabolism of dibenzothiophene by a Beijerinckia species. Appl. Environ. Microbiol..

[102] Khan A.A., Wang R.F., Cao W.W., Franklin W., Cerniglia C.E. (1996). Reclassification of a polycyclic aromatic hydrocarbon-metabolizing bacterium, Beijerinckia sp strain B1, as Sphingomonas yanoikuyae by fatty acid analysis, protein pattern analysis, DNA-DNA hybridization, and 16S ribosomal DNA sequencing. Int. J. System. Bacteriol..

[103] Bianchi D., Bosetti A., Cidaria D., Bernardi A., Gagliardi I., D’Amico P. (1997). Oxidation of polycyclic aromatic heterocycles by Pseudomonas fluorescens TTC1. Appl. Microbiol. Biotechnol..

[104] Saftic S., Fedorak P.M., Andersson J.T. (1993). Transformations of methyldibenzothiophenes by three Pseudomonas isolates. Environ. Sci. Technol..

[105] Gibson D.T., Resnick S.M., Lee K., Brand J.M., Torok D.S., Wackett L.P., Schocken M.J., Haigler B.E. (1995). Desaturation, dioxygenation, and monooxygenation reactions catalyzed by naphthalene dioxygenase from Pseudomonas SP strain-9816–4. J. Bacteriol..

[106] Resnick S., Lee K., Gibson D.T. (1996). Diverse reactions catalyzed by naphthalene dioxygenase fromPseudomonas sp strain NCIB 9816. J. Ind. Microbiol. Biotechnol..

[107] Eaton S.L., Resnick S.M., Gibson D.T. (1996). Initial reactions in the oxidation of 1,2-dihydronaphthalene by Sphingomonas yanoikuyae strains. Appl. Environ. Microbiol..

[108] Misra B., Amin S. (1990). Synthesis and mutagenicity of trans-dihydrodiol metabolites of benzo[b]naphtho[2,1-d]thiophene. Chem. Res. Toxicol..

[109] Murphy S.E., Amin S., Coletta K., Hoffmann D. (1992). Rat liver metabolism of benzo[b]naphtho[2,1-d]thiophene. Chem. Res. Toxicol..

[110] Boyd D.R., Sharma N.D., Sbircea L., Murphy D., Belhocine T., Malone J.F., James S.L., Allen C.C.R., Hamilton J.T.G. (2008). Azaarene cis-dihydrodiol-derived 2,2′-bipyridine ligands for asymmetric allylic oxidation and cyclopropanation. Chem. Commun..

[111] Boyd D.R., Sharma N.D., Sbircea L., Murphy D., Malone J.F., James S.L., Allen C.C.R., Hamilton J.T.G. (2010). Chemoenzymatic synthesis of chiral 2,2′-bipyridine ligands and their N-oxide derivatives: Applications in the asymmetric aminolysis of epoxides and asymmetric allylation of aldehydes. Org. Biomol. Chem..

[112] Sbircea L., Sharma N.D., Clegg W., Harrington R.W., Horton P.N., Hursthouse M.B., Apperley D.C., Boyd D.R., James S.L. (2008). Chemoenzymatic synthesis of chiral 4,4′-bipyridyls and their metal–organic frameworks. Chem. Commun..

[113] Nakayama J., Tajima Y., Xue-Hua P., Sugihara Y. (2007). [1 + 2] Cycloadditions of Sulfur Monoxide (SO) to Alkenes and Alkynes and [1 + 4] Cycloadditions to Dienes (Polyenes). Generation and Reactions of Singlet SO?. J. Am. Chem. Soc..

[114] Dansette P., Jerina D.M. (1974). Facile synthesis of arene oxides at the K regions of polycyclic hydrocarbons. J. Am. Chem. Soc..

[115] Mitchell J.R., Jollows D.J. (1975). Metabolic Activation of Drugs to Toxic Substances. Gastroenterology.

[116] McMurtry R., Mitchell J. (1977). Renal and hepatic necrosis after metabolic activation of 2-substituted furans and thiophenes, including furosemide and cephaloridine. Toxicol. Appl. Pharmacol..

[117] Dansette P.M., Thang D.C., ElAmri H., Mansuy D. (1992). Evidence for thiophene-S-oxide as primary reactive metabolite of thiophene in vivo- formation of a dihydrothiophene sulfoxide mercapturic acid. Biochem. Biophys. Res. Commun..

[118] Li Y., Thiemann T., Sawada T., Mataka S., Tashiro M. (1997). Lewis Acid Catalysis in the Oxidative Cycloaddition of Thiophenes1. J. Org. Chem..

[119] Sodhi J.K., Delarosa E.M., Halladay J.S., Driscoll J.P., Mulder T., Dansette P.M., Khojasteh S.C. (2017). Inhibitory Effects of Trapping Agents of Sulfur Drug Reactive Intermediates against Major Human Cytochrome P450 Isoforms. Int. J. Mol. Sci..

[120] Dansette P.M., Thebault S., Durand-Gasselin L., Bertho G., Mansuy D. (2010). Reactive metabolites of thiophenic compounds: A new trapping method for thiophene sulfoxides. Drug Metab. Rev..

[121] Du F., Ruan Q., Zhu M., Xing J. (2013). Detection and characterization of ticlopidine conjugates in rat bile using high-resolution mass spectrometry: Applications of various data acquisition and processing tools. J. Mass Spectrom..

[122] Jaladanki C.K., Taxak N., Varikoti R.A., Bharatam P.V. (2015). Toxicity Originating from Thiophene Containing Drugs: Exploring the Mechanism using Quantum Chemical Methods. Chem. Res. Toxicol..

[123] Imami A., Herold N., Spielmeyer A., Hausmann H., Dötzer R., Behnken H.N., Leonhardt S., Weil A., Schoof S., Zorn H. (2016). Biotransformation of Dimethenamid-P by the basidiomycete Irpex consors. Chemosphere.

[124] Jerina D.M., Daly J.W., Witkop B., Zaltzman-Nirenberg P., Udenfriend S. (1968). The role of arene oxide-oxepin systems in the metabolism of aromatic substrates. III. Formation of 1,2-naphthalene oxide from naphthalene by liver microsomes. J. Am. Chem. Soc..

[125] Jerina D.M., Daly J.W. (1974). Arene Oxides: A New Aspect of Drug Metabolism. Science.

[126] Mansuy D., Dansette P.M., Foures C., Jaouen M., Moinet G., Bayer N. (1984). Metabolic hydroxylation of the thiophene ring: Isolation of 5-hydroxy-tienilic acid as the major urinary metabolite of tienilic acid in man and rat. Biochem. Pharmacol..

[127] Dalvie D.K., Kalgutkar A.S., Khojasteh-Bakht S.C., Obach R.S., O’Donnell J.P. (2002). Biotransformation Reactions of Five-Membered Aromatic Heterocyclic Rings. Chem. Res. Toxicol..

[128] Belghazi M., Jean P., Poli S., Schmitter J.M., Mansuy D., Dansette P.M., Dansette P.M., Snyder R., Delaforge M., Gibson G.G., Greim H., Jollow D.J., Monks T.J., Sipes I.G. (2001). Use of isotopes and LC-MS-ESI-TOF for mechanistic studies of Tienilic Acid metabolic activation. Biological Reactive Intermediates VI.

[129] Mori Y., Sakai Y., Kuroda N., Yokoya F., Toyoshi K., Horie M., Baba S. (1984). Further structural analysis of urinary metabolites of suprofen in the rat. Drug Metab. Dispos..

[130] Fouda H.G., Avery M.J., Dalvie D., Falkner F.C., Melvin L.S., Ronfeld R.A. (1997). Disposition and metabolism of tenidap in the rat. Drug Metab. Dispos..

[131] Neau E., Dansette P., Andronik V., Mansuy D. (1990). Hydroxylation of the thiophene ring by hepatic monooxygenases: Evidence for 5-hydroxylation of 2-aroylthiophenes as a general metabolic pathway using a simple UV-visible assay. Biochem. Pharmacol..

[132] Nishikawa T., Nagata O., Tanbo K., Yamada T., Takahara Y., Kato H., Yamamoto Y. (1985). Absorption, excretion and metabolism of tiquizium bromide in dogs, and relationship between pharmacological effect and plasma levels of unchanged drug. Xenobiotica.

[133] Lynch M.J., Mosher F.R., Levesque W.R., Newby T.J. (1987). The In Vitro and In Vivo Metabolism of Morantel in Cattle and Toxicology Species. Drug Metab. Rev..

[134] Ichihara S., Tsuyuki Y., Tomisawa H., Fukazawa H., Nakayama N., Tateishi M., Joly R. (1984). Metabolism of tenoxicam in rats. Xenobiotica.

[135] Boyd D.R., Sharma N.D. (1996). The changing face of arene oxide–oxepine chemistry. Chem. Soc. Rev..

[136] Barr S.A., Bowers N., Boyd D.R., Sharma N.D., Hamilton L., Austin R., McMordie R.A.S., Dalton H. (1998). The potential role of cis-dihydrodiol intermediates in bacterial aromatic hydroxylation and the NIH Shift. J. Chem. Soc. Perkin Trans..

[137] Stok J.E., Chow S., Krenske E.H., Soto C.F., Matyas C., Poirier R.A., Williams C.M., De Voss J.J. (2016). Direct Observation of an Oxepin from a Bacterial Cytochrome P450-Catalyzed Oxidation. Chem.—Eur. J..

[138] Boyd D.R., Sharma N.D., Harrison J.S., Malone J.F., McRoberts W.C., Hamilton J.T.G., Harper D.B. (2008). Enzyme-catalysed synthesis and reactions of benzene oxide/oxepine derivatives of methyl benzoates. Org. Biomol. Chem..

[139] Taguchi K., Konishi T., Nishikawa H., Kitamura S. (1999). Identification of human cytochrome P450 isoforms involved in the metabolism ofS-2-[4-(3-methyl-2-thienyl)phenyl]propionic acid. Xenobiotica.

[140] Dansette P.M., Kitamura S., Bertho G., Mansuy D. (2005). A new thiophene metabolite: NADPH, O2 and thiol dependent formation of a thiophene hydrate from (S) MTPPA (methyl-thienyl-phenyl-propionic acid) by CYP2C9. Drug Metab. Rev..

[141] Boyd D.R., Sharma N.D., Agarwal R., McMordie R.A.S., Bessems J., van Ommen B., van Bladeren P.J. (1993). Biotransformation of 1,2-dihydronaphthalene and 1,2-dihydroanthracene by rat liver microsomes and purified cytochromes P-450. Formation of arene hydrates of naphthalene and anthracene. Chem. Res. Toxicol..

[142] Lopez-Garcia M.P., Dansette P.M., Mansuy D. (1994). Thiophene derivatives as new mechanism-based inhibitors of cytochromes P-450: Inactivation of yeast-expressed human liver cytochrome P-450 2C9 by tienilic acid. Biochemistry.

[143] Ha-Duong N.-T., Dijols S., Macherey A.-C., Goldstein J.A., Dansette A.P.M., Mansuy D. (2001). Ticlopidine as a Selective Mechanism-Based Inhibitor of Human Cytochrome P450 2C19. Biochemistry.

[144] Richter T., Mürdter T.E., Heinkele G., Pleiss J., Tatzel S., Schwab M., Eichelbaum M., Zanger U.M. (2003). Potent Mechanism-Based Inhibition of Human CYP2B6 by Clopidogrel and Ticlopidine. J. Pharmacol. Exp. Ther..

[145] Hutzler J.M., Balogh L.M., Zientek M., Kumar V., Tracy T.S. (2008). Mechanism-Based Inactivation of Cytochrome P450 2C9 by Tienilic Acid and (±)-Suprofen: A Comparison of Kinetics and Probe Substrate Selection. Drug Metab. Dispos..

[146] Lin H.-L., Zhang H., Medower C., Hollenberg P.F., Johnson W.W. (2010). Inactivation of Cytochrome P450 (P450) 3A4 but not P450 3A5 by OSI-930, a Thiophene-Containing Anticancer Drug. Drug Metab. Dispos..

[147] Kalgutkar A.S., Driscoll J., Zhao S.X., Walker G.S., Shepard R.M., Soglia J.R., Atherton J., Yu L., Mutlib A.E., Munchhof M.J. (2007). A Rational Chemical Intervention Strategy To Circumvent Bioactivation Liabilities Associated with a Nonpeptidyl Thrombopoietin Receptor Agonist Containing a 2-Amino-4-arylthiazole Motif. Chem. Res. Toxicol..

[148] Barnette D., Schleiff M., Osborn L.R., Flynn N., Matlock M., Swamidass S.J., Miller G.P. (2020). Dual mechanisms suppress meloxicam bioactivation relative to sudoxicam. Toxicology.

[149] Obach R.S., Kalgutkar A.S., Ryder T.F., Walker G.S. (2008). In Vitro Metabolism and Covalent Binding of Enol-Carboxamide Derivatives and Anti-Inflammatory Agents Sudoxicam and Meloxicam: Insights into the Hepatotoxicity of Sudoxicam. Chem. Res. Toxicol..

[150] Nikodinovic-Runic J., Coulombel L., Francuski D., Sharma N.D., Boyd D.R., More O’Ferrall R.A., O’Connor K.E. (2013). The oxidation of alkylaryl sulfides and benzo[b]thiophenes by Escherichia coli cells expressing wild-type and engineered styrene monooxygenase from Pseudomonas putida CA-3. Appl. Microbiol. Biotechnol..

[151] Gursky L.J., Nikodinović-Runić J., Feenstra K.A., O’Connor K.E. (2010). In vitro evolution of styrene monooxygenase from Pseudomonas putida CA-3 for improved epoxide synthesis. Appl. Microbiol. Biotechnol..

[152] O’Connor K.E., Dobson A.D., Hartmans S. (1997). Indigo formation by microorganisms expressing styrene monooxygenase activity. Appl. Environ. Microbiol..

[153] Chen B., Zhang X.-D., Wen J., Zhang B., Chen D., Wang S., Cai J.-P., Hu G.-X. (2020). Effects of 26 Recombinant CYP3A4 Variants on Brexpiprazole Metabolism. Chem. Res. Toxicol..

[154] Joshi E.M., Heasley B.H., Chordia M., Macdonald T.L. (2004). In Vitro Metabolism of 2-Acetylbenzothiophene: Relevance to Zileuton Hepatotoxicity. Chem. Res. Toxicol..

[155] Parker M.H., Smith-Swintosky V.L., McComsey D.F., Huang Y., Brenneman D., Klein B., Malatynska E., White H.S., Milewski M.E., Herb M. (2009). Novel, Broad-Spectrum Anticonvulsants Containing a Sulfamide Group: Advancement of N-((Benzo[b]thien-3-yl)methyl)sulfamide (JNJ-26990990) into Human Clinical Studies. J. Med. Chem..

[156] Jacob J., Schmoldt A., Grimmer G. (1986). The predominant role of S-oxidation in rat liver metabolism of thiaarenes. Cancer Lett..

[157] Hollopeter G., Jantzen H.-M., Vincent D., Li G., England L., Ramakrishnan V., Yang R.-B., Nurden P., Nurden A., Julius D. (2001). Identification of the platelet ADP receptor targeted by antithrombotic drugs. Nature.

[158] Maffrand J.-P. (2012). The story of clopidogrel and its predecessor, ticlopidine: Could these major antiplatelet and antithrombotic drugs be discovered and developed today?. Comptes Rendus Chim..

[159] Kiebist J., Schmidtke K.-U., Schramm M., König R., Quint S., Kohlmann J., Zuhse R., Ullrich R., Hofrichter M., Scheibner K. (2021). Biocatalytic Syntheses of Antiplatelet Metabolites of the Thienopyridines Clopidogrel and Prasugrel Using Fungal Peroxygenases. J. Fungi.

[160] Dansette P.M., Libraire J., Bertho G., Mansuy D. (2009). Metabolic Oxidative Cleavage of Thioesters: Evidence for the Formation of Sulfenic Acid Intermediates in the Bioactivation of the Antithrombotic Prodrugs Ticlopidine and Clopidogrel. Chem. Res. Toxicol..

[161] Dansette P.M., Thébault S., Bertho G., Mansuy D. (2010). Formation and Fate of a Sulfenic Acid Intermediate in the Metabolic Activation of the Antithrombotic Prodrug Prasugrel. Chem. Res. Toxicol..

[162] Dansette P.M., Rosi J., Bertho G., Mansuy D. (2011). Paraoxonase-1 and clopidogrel efficacy. Nat. Med..

[163] Dansette P.M., Rosi J., Bertho G., Mansuy D. (2012). Cytochromes P450 Catalyze Both Steps of the Major Pathway of Clopidogrel Bioactivation, whereas Paraoxonase Catalyzes the Formation of a Minor Thiol Metabolite Isomer. Chem. Res. Toxicol..

[164] Dansette P.M., Levent D., Hessani A., Bertho G., Mansuy D. (2013). Thiolactone Sulfoxides as New Reactive Metabolites Acting as Bis-Electrophiles: Implication in Clopidogrel and Prasugrel Bioactivation. Chem. Res. Toxicol..

[165] Zhang H., Lau W.C., Hollenberg P.F. (2012). Formation of the thiol conjugates and active metabolite of clopidogrel by human liver microsomes. Mol. Pharmacol..

[166] Tuong A., Bouyssou A., Paret J., Cuong T.G. (1981). Metabolism of triclopidine in rats: Identification and quantitative determination of some its metabolites in plasma, urine and bile. Eur. J. Drug Metab. Pharmacokinet..

[167] Farid N.A., Smith R.L., Gillespie T.A., Rash T.J., Blair P.E., Kurihara A., Goldberg M.J. (2007). The Disposition of Prasugrel, a Novel Thienopyridine, in Humans. Drug Metab. Dispos..

[168] Liu C., Chen Z., Zhong K., Li L., Zhu W., Chen X., Zhong D. (2015). Human Liver Cytochrome P450 Enzymes and Microsomal Thiol Methyltransferase Are Involved in the Stereoselective Formation and Methylation of the Pharmacologically Active Metabolite of Clopidogrel. Drug Metab. Dispos..

[169] Kazui M., Hagihara K., Izumi T., Ikeda T., Kurihara A. (2014). Hepatic Microsomal Thiol Methyltransferase Is Involved in Stereoselective Methylation of Pharmacologically Active Metabolite of Prasugrel. Drug Metab. Dispos..

[170] Camilleri P., Soldo B., Buch A., Janusz J. (2021). Oxidative metabolism of razuprotafib (AKB-9778), a sulfamic acid phosphatase inhibitor, in human microsomes and recombinant human CYP2C8 enzyme. Xenobiotica.

[171] Soldo B.L., Camilleri P., Buch A., Janusz J. (2021). The in vivo disposition of subcutaneous injected 14C-razuprotafib (14C-AKB-9778), a sulphamic acid phosphatase inhibitor, in nonclinical species and human. Xenobiotica.

[172] Guengerich F.P. (2020). A history of the roles of cytochrome P450 enzymes in the toxicity of drugs. Toxicol. Res..

[173] Shimizu S., Atsumi R., Nakazawa T., Fujimaki Y., Sudo K., Okazaki O. (2009). Metabolism of Ticlopidine in Rats: Identification of the Main Biliary Metabolite as a Glutathione Conjugate of Ticlopidine S-Oxide. Drug Metab. Dispos..

